# Characterization of human transcription factor function and patterns of gene regulation in HepG2 cells

**DOI:** 10.1101/gr.278205.123

**Published:** 2023-11

**Authors:** Belle A. Moyers, E. Christopher Partridge, Mark Mackiewicz, Michael J. Betti, Roshan Darji, Sarah K. Meadows, Kimberly M. Newberry, Laurel A. Brandsmeier, Barbara J. Wold, Eric M. Mendenhall, Richard M. Myers

**Affiliations:** 1 HudsonAlpha Institute for Biotechnology, Huntsville, Alabama 35806, USA;; 2 Vanderbilt University Medical Center, Nashville, Tennessee 37232, USA;; 3 Merkin Institute for Translational Research, California Institute of Technology, Pasadena, California 91125, USA

## Abstract

Transcription factors (TFs) are *trans*-acting proteins that bind *cis*-regulatory elements (CREs) in DNA to control gene expression. Here, we analyzed the genomic localization profiles of 529 sequence-specific TFs and 151 cofactors and chromatin regulators in the human cancer cell line HepG2, for a total of 680 broadly termed DNA-associated proteins (DAPs). We used this deep collection to model each TF's impact on gene expression, and identified a cohort of 26 candidate transcriptional repressors. We examine high occupancy target (HOT) sites in the context of three-dimensional genome organization and show biased motif placement in distal-promoter connections involving HOT sites. We also found a substantial number of closed chromatin regions with multiple DAPs bound, and explored their properties, finding that a MAFF/MAFK TF pair correlates with transcriptional repression. Altogether, these analyses provide novel insights into the regulatory logic of the human cell line HepG2 genome and show the usefulness of large genomic analyses for elucidation of individual TF functions.

Gene expression is regulated and modulated by the association, either direct or indirect, of various classes of proteins to DNA, including RNA polymerase and transcription-associated proteins, histone modifiers, and a broad suite of transcription factors (TFs) and associated cofactors. Together, these DNA-associated proteins (DAPs) are encoded by ∼10% of all protein-coding genes in the human genome (Vaquerizas et al. 2009; Lambert et al. 2018). DAPs are known to associate with DNA either through recognition of discrete small sequence motifs, by interactions with degenerate sequences having little complexity, or by cofactor recruitment. The most common assay for genome-wide identification of genomic binding or association sites for DAPs is chromatin immunoprecipitation followed by high-throughput sequencing (ChIP-seq), which provides a statistically identified snapshot of regions referred to as peaks (Barski et al. 2007; Johnson et al. 2007; Robertson et al. 2007; Kharchenko et al. 2008; Zhang et al. 2008; Savic et al. 2015; Meadows et al. 2020). For those TFs with DNA sequence specificity, associations occur with enough frequency to be detectable as a consistent DNA sequence motif through use of genome-wide binding data (Bailey et al. 2015) or in vitro molecular binding assays (Chai et al. 2011).

The Encyclopedia of DNA Elements (ENCODE) Consortium has completed and released 3194 ChIP-seq data sets for 1139 DAPs using both traditional antibody ChIP-seq and epitope-tagged ChIP-seq methods (The ENCODE Project Consortium 2012; The ENCODE Project Consortium et al. 2020; Partridge et al. 2020). The human liver cancer–derived cell line HepG2 currently has the largest number (n = 814) of ENCODE-released ChIP-seq data sets, some of which are repetitions of different ChIP-seq experiments with the same target for a total of 680 unique DAP targets. With this wealth of occupancy profiles for a single cell type, the HepG2 ChIP-seq data allow for the assessment of biological roles of DAPs in a broad genomic context, including analyses of similarity and coassociation frequency, association with regulatory region types, and impact on gene expression. These data sets provide the opportunity to explore the functional impact of individual TFs and associated proteins on gene expression and genome organization.

Here, we present an analysis of ChIP-seq data in the HepG2 that greatly expands on our previous work with this cell type (Partridge et al. 2020), including 492 ChIP-seq data sets not analyzed in that prior work, as well as a lentiviral massively parallel reporter assay (lentiMPRA, or MPRA) to functionally test elements. We provide an overview of this resource and highlight novel findings with TFs and *trans*-regulatory proteins on *cis*-regulatory sequences, including patterns of TF genomic localization in the context of the three-dimensional (3D) organization of high occupancy target (HOT) sites and the association of TFs with closed chromatin regions that influence gene repression.

## Results

It is estimated that there are 1639 sequence-specific TFs encoded in the human genome (Lambert et al. 2018), only a subset of which are expressed in any given cell type. To gain a deeper understanding of gene regulatory mechanisms, we analyzed TF binding data, much of which we generated, in HepG2 cells and leveraged the large number of TFs assayed in that cell line as the most comprehensive resource available. The expression level of any individual TF is not necessarily correlated with its biological significance; proteins can be expressed at a very low level and still perform important biological functions in a given context. Pragmatically, however, we have observed diminishing rates of success for ChIP-seq and epitope-tagged ChIP-seq data sets as the expression level of those TFs decreases (Meadows et al. 2020). Therefore, we identified all TFs in HepG2 cells that are expressed at levels of at least two transcripts per million (TPM), as measured by RNA-seq (ENCSR181ZGR). There are 895 TFs expressed at this level in HepG2 cells. We compiled the existing data sets produced from our laboratory and others from the ENCODE portal for 479 (53.5%) of these 895 TFs (see Methods) (Supplemental Table 1). In addition to these 479 TFs, we also analyzed data for 50 TFs expressed at fewer than two TPM for which we were able to generate high-quality ChIP-seq data despite their low expression and for 151 non-TF DAPs and nine histone marks, for a total of 680 unique ChIP-seq DAP targets in HepG2 and nine histone modifications. This expanded catalog of DAPs and associated gene regulatory data sets provides a rich resource to characterize and understand the functional impact of DAP binding on gene regulation.

### DAP associations at cCREs reveal the interaction of DAP function and regulatory context

**Figure 1.**
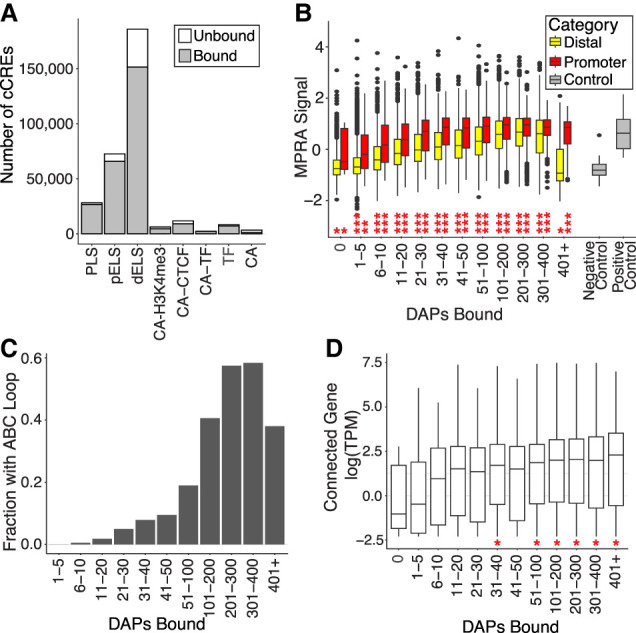
Genomic properties and activities of DAP-bound regions in genomic and reporter contexts. (*A*) The majority of cCREs of each type are bound by an assayed DAP. Bars show the number of sites of each cCRE class (*x*-axis) with at least one DAP association (“bound”) and those with none in our data set (“unbound”) when restricted to those overlapping with an ATAC-seq peak in HepG2. (PLS) Promoter-like signature, (pELS) proximal enhancer-like signature, (dELS) distal enhancer-like signature, (CA-H3K4me3) chromatin-accessible H3K4me3 region, (CA-CTCF) chromatin-accessible CTCF-bound region, (CA-TF) chromatin-accessible TF-bound region, (TF) TF-bound region lacking chromatin accessibility, and (CA) chromatin accessibility only. (*B*) Promoter elements from locally performed lentiMPRA experiments require fewer DAPs binding for high activity in lentiMPRA than do distal elements. Boxes show MPRA signal (natural log of normalized RNA reads over normalized DNA reads) of promoter elements as a function of binned number of DAPs (*x*-axis) with a peak in the genomic region. Promoters are defined as elements whose bounds overlapped with a 200-bp region centered on GENCODE TSSs. Distal elements are defined as elements at least 5 kb from annotated TSSs. Positive and negative control elements are plotted for comparison. In *B* and *D*, boxes represent 25%–75% quartiles with lines indicating the median, whiskers extend to ±1.5 × IQR (interquartile range) past the boxes, and when present, points are observations falling outside of this range. Unpaired *t*-tests were used to identify significant differences in the means between distal and promoter element activity in each category. (*) *P* = 0.05, (**) *P* = 0.0001, (***) *P* ≤ 2.2 × 10^−16^. (*C*) The fraction of distal loci with an ABC connection as a function of binned number of DAPs at a distal element. (*D*) Expression of genes genome-wide increases as the number of factors bound and connected distal elements increases. The *y*-axis indicates the natural log expression distribution of the ABC-supported gene as a function of binned number of DAPs at a distal element. Unpaired *t*-tests were used to identify significant differences in the means between the expression of a given category compared with expression in the zero category. (*) *P* = 0.05.

TFs impact expression by associating with or binding to DNA, specifically at *cis*-regulatory elements (CREs). We therefore sought to determine which *cis*-regulatory elements are bound by TFs and the patterns of activity that those bound regions display. We examined *cis*-regulatory elements for the presence of at least one DAP peak. To do this, we used the Registry of Candidate *cis*-Regulatory Elements (V4 cCREs) derived from the ENCODE data (The ENCODE Project Consortium et al. 2020; JE Moore, HE Pratt, K Fan, et al., in prep.). These candidate *cis-*regulatory elements (cCREs) represent genomic regulatory elements across multiple human cell types and are derived from chromatin accessibility assays (DNase-seq and ATAC-seq), histone modifications, and DAP-binding data. To filter for cCREs that are relevant in HepG2, we overlapped with HepG2 ATAC-seq data, generating a set of 318,567 HepG2 cCREs. Of these, 84.2% have at least one of the assayed DAPs associated, and those cCREs with no DAPs associated in HepG2 are largely distal enhancer-like sequences (Fig. 1A; Supplemental Fig. 1; Supplemental Table 2). We compared this pattern of binding with dinucleotide-matched control sequences and found that these regions are significantly more bound than controls (Supplemental Fig. 2; Supplemental Table 2). As the number of associated DAPs increases at cCREs, the proportion of cCREs defined as “promoter-like” increases (Supplemental Figs. 3, 4; Supplemental Table 3). We therefore conclude that the coverage of cCREs with at least some subset of their associated DAPs is approaching completeness.

We also found 50,446 (15.8%) annotated cCREs overlapping with an ATAC-seq peak in HepG2 cells but with no DAP peaks in our data set. Given their predicted regulatory activity and their open chromatin state in this cell type, we would expect that they should be bound by some DAP. At least three explanations are possible: (1) These cCREs are unbound by any DAP, (2) they are bound by DAPs that have not yet been assayed in HepG2 cells, and/or (3) DAP binding was potentially missed as false negatives in the ChIP-seq assays. To measure functional activity of these elements (as well as for other analyses below), we performed a lentiMPRA (or MPRA) following established methods (Gordon et al. 2020). MPRAs functionally validate the regulatory activity of thousands of DNA elements simultaneously by insertion of DNA upstream of or downstream from a transcribed element (Klein et al. 2020). Our MPRA experiment contained 69,210 elements of 170 bp each, selected from various promoter and distal cCREs and from non-cCREs, as well as a set of synthetic, nongenomic elements with various numbers of TF motifs. We supplemented this data set by also analyzing a publicly available HepG2 lentiMPRA data set containing 139,877 elements of 200 bp each (Agarwal et al. 2023), representing all cCREs published under the previous ENCODE cCRE version 3 release. We found that only 32% of the elements in our lentiMPRA had any overlap with elements in the Agarwal MPRA, indicating that these assays provided substantially different information. In both of these lentiMPRA data sets, the cCREs without DAP peaks had, on average, lower MPRA signal than did the elements with DAP peaks. However, these elements had significantly higher activity than negative control elements in our MPRA (Fig. 1B; Supplemental Table 4), suggesting that some of these regions have regulatory activity and presumably have DAP associations that are not present in our data set. We confirmed that this finding remained true using the Agarwal lentiMPRA (Supplemental Fig. 5; Supplemental Table 5). To estimate how much additional cCRE coverage we are missing from the more than 400 DAPs expressed at a TPM of two or more in HepG2 cells but that have not yet been assayed, we performed a subsampling analysis to determine how the fraction of the cCREs covered changes with varying numbers of analyzed DAPs. We note that the first 200 DAPs appear to add a large amount of information, but for new DAPs beyond 200, there is a sharp decrease in new coverage. We extrapolate that assaying an additional 400 DAPs would result in total coverage of only 88%–90% of HepG2 cCREs (Supplemental Fig. 6). This suggests that adding more DAP data sets generated with ChIP-seq would not result in complete coverage of all HepG2 cCREs and may potentially be insufficient to detect *trans*-regulatory factors at some fraction of these sites. To further explore whether peak-calling in ChIP-seq misses some meaningful DAP associations, we built a gkm-SVM model (Ghandi et al. 2016) for each DAP based on its bound sequences and applied these models to unbound regions. We found that 67% of cCRE regions without DAP peak calls had a strong score for at least one DAP's gkm-SVM model (Supplemental Fig. 7; Supplemental Table 6), suggesting that there are missed associations at these genomic regions. Finally, to explore this further, we used a sample of 45 bigWig signal files from the ENCODE Project to determine whether the signal over these regions is meaningfully different. We found that in 51.1% of these bigWig files, unbound cCREs with gkm-SVM scores at or above the 90th percentile had a higher signal than unbound regions with gkm-SVM scores at or below the 10th percentile (Supplemental Fig. 8). This suggests that many of these regions may represent false-negative peak calls for at least one TF. As an alternative to ChIP-seq, predictive computational methods for DAP binding may offer further insights (Ghandi et al. 2016; Schreiber et al. 2020).

We asked how DAPs work together and impact gene expression at different cCREs, specifically promoters and distal elements. As a measure of the functional impact of DAP associations, we assessed how varying numbers of DAP associations at cCREs impact potential gene expression by examining the correlation between numbers of DAPs and transcriptional activity in our lentiviral MPRA experiment (Fig. 1B; Supplemental Table 4) and the MPRA from Agarwal et al. (2023) (Supplemental Fig. 5; Supplemental Table 5). We observed a trend in both promoters and distal elements in which expression increased as the number of bound DAPs increased. This supports earlier findings that MPRA activity correlates with the number of DAPs bound at the endogenous element (Ramaker et al. 2020). Elements from distal regions have a much wider distribution of expression but, on average, lower levels of reporter expression compared with promoters for each bin of DAP numbers. This is expected in the lentiMPRA assay system, in which test elements are directly upstream of the reporter open reading frame and thus ideal for promoter activity tests. However, distal elements with very large numbers of associated DAPs (more than 400) showed a sharp decrease in activity, whereas promoter elements with these numbers did not. As this is a novel finding, we confirmed the results by using a second MPRA data set (Supplemental Fig. 5; Supplemental Table 5). Thus, distal elements with a high number of TFs and associated cofactors show significantly lower activity in MPRA assays versus elements with fewer DAPs bound.

To further explore the distal elements with high numbers of DAPs yet low expression in the MPRA and to uncover potential explanations for this finding, we examined these regions more broadly. We speculated that placing these regions in an artificial context close to the promoter of a reporter gene may not accurately recapitulate their endogenous looping to distal promoters to enhance gene expression. Therefore, we examined activity-by-contact (ABC) loop models (Fulco et al. 2019) in HepG2, an analysis that assigns a confidence score to an active loop, or a connection between a promoter and a distal element using 3D interaction metrics in the form of Hi-C data, as well as RNA-seq, ATAC-seq, and histone marks. Crucially, the ABC method does not consider DAP binding as a contribution to the score. We found that distal elements are increasingly likely to have an ABC connection to at least one promoter as the number of DAPs increases until reaching about 200 DAPs, with a substantial drop in connections for elements with more than 400 associated DAPs (Fig. 1C). We compared this with dinucleotide-matched control sequences and found that, compared with controls, these regions are enriched for ABC associations (Supplemental Fig. 9; Supplemental Table 7). Nonetheless, we found that very highly bound distal elements that do contain an ABC loop tended to be connected to highly expressed genes (Fig. 1D; Supplemental Table 8). This suggests that these elements functionally enhance gene expression but not in a proximal MPRA context, and highlights the importance of regulatory context on DAP function. To explore these regions further, we identified all bound regions in the genome with 201–400 DAPs bound and compared them with regions with 401+ DAPs bound. We found that regions with 401+ DAPs bound were, on average, larger (mean, 1703 bp vs. 1237 for 201–400); have higher GC content (59.1% vs. 57.9%, *P* = 8.65 × 10^−4^, Mann–Whitney *U* test); and have higher ATAC-seq signal (mean bigWig signal 538.4 vs. 443.75, *P* ≤ 2.2 × 10^−16^, Mann–Whitney *U* test) (Supplemental Fig. 10).

### Modeling of TF effects on gene expression identifies putative *trans*-activators and repressors

**Figure 2.**
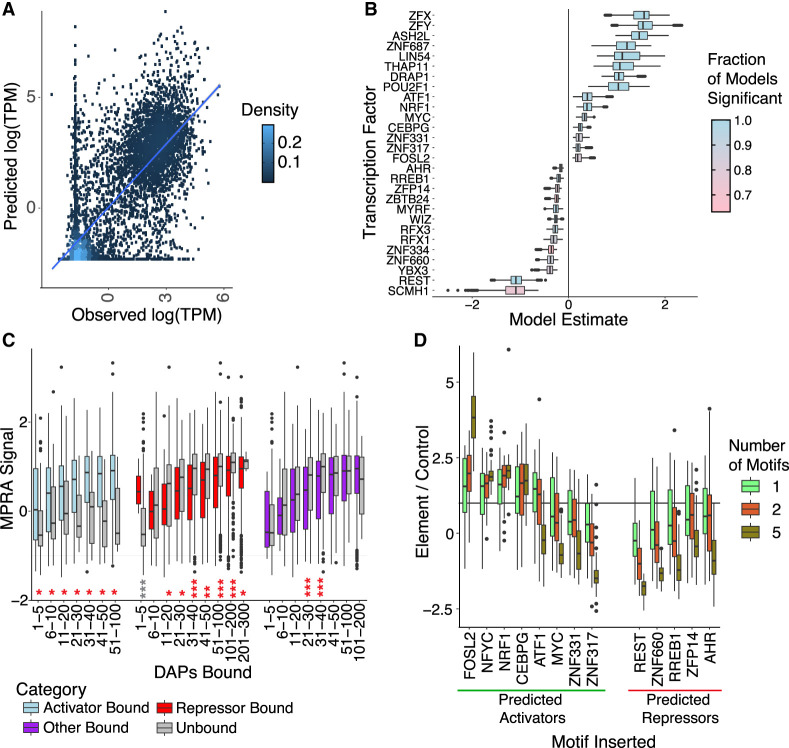
Effects of specific TFs on gene expression and in MPRA assays. (*A*) Observed (*x*-axis) versus predicted (*y*-axis) natural log of gene expression as measured by transcripts per million (TPM). A linear model was constructed based on binding of TFs at a gene's TSS ±500 bp. Training and testing were performed on a 70%/30% split of all genes. Pearson's correlation = 0.77, *P* ≤ 2.2 × 10^−16^. Blue line was generated from geom_smooth in the ggplot2 package (Wickham 2009). (*B*) Box plot shows distribution of linear model estimates (*x*-axis) for select TFs (*y*-axis) from submodels. Five hundred submodels with unique subsets of randomized TFs (*n* = 79) were constructed for each TF, and estimates for the focal TF were recorded. Colors closer to blue indicate that the focal TF was significant in a higher proportion of submodels, and colors closer to pink indicate that the focal TF was significant in a lower proportion of submodels. For *B*–*D*, boxes represent 25%–75% quartiles with line indicating median, whiskers extend to ±1.5 × IQR (interquartile range) past the boxes, and points are observations falling outside of this range. (*C*) Boxes show MPRA signal (natural log of normalized RNA reads over normalized DNA reads; *y*-axis) as a function of binned number of DAPs (*x*-axis) for promoter regions either bound by one of the top factors identified in the linear model as an activator (blue), repressor (red) or randomly selected TF (purple), compared with regions that were not bound by one of those TFs for each group (gray), showing activating, repressing, and uncertain activity for each respective group of TFs, respectively. Unpaired *t*-tests were used to identify significant differences in the means between bound and unbound sequences in each group. (*) *P* = 0.05, (**) *P* = 0.0001, (***) *P* ≤ 2.2 × 10^−16^. (*D*) Boxes show MPRA signal as in *C* (*y*-axis) for motifs inserted into enhancer sequences at various intervals (*x*-axis). A group of candidate activators (*x*-axis; green line) and candidate repressors (*x*-axis; red line) was selected, and one (green), two (red), or five (brown) motifs were inserted. Control ratio was based on the sequence without any motif insertions. *P*-values for this figure are available in Supplemental Table 16.

We next asked whether these numerous ChIP-seq data sets would allow us to predict and quantify each TF's activating or repressing behavior. TFs are a specific subset of DAPs that bind to DNA in a sequence-specific manner to regulate transcription (Lambert et al. 2018). Parsing out specific contributions of TFs to expression patterns is an ongoing effort in the genomics community. To explore functional effects of TF localization on gene expression, we created models based on the association of 529 TFs to the promoter region of genes. Linear modeling of gene expression based on promoter TF association offers a clear and interpretable effect of each TF as activating or repressing. Our reasoning for limiting this analysis to the promoter regions of annotated genes, as opposed to including all distal candidate enhancers, was to cleanly assign the TF binding event to a gene expression outcome. We built linear models based on 70% of promoters and used these to predict gene expression on the remaining 30% of promoters. We achieved accurate correlation between predictions and observation (Fig. 2A), comparable with neural network models based on large amounts of sequence data (Agarwal and Shendure 2020). Prediction accuracy was robust to promoter subtypes (CpG island [CGI] promoters and non-CGI promoters) and was applicable across cell types (Supplemental Fig. 11–13). Prediction accuracy was also moderately better than estimating expression levels on the number of associated TFs alone (Supplemental Fig. 14). Although we note that there are some cases in which observed expression is high and predicted expression is low, as well as the converse, these are a minority of cases (∼10%) and likely represent either the effects of factors that were not included in our ChIP-seq data sets, the role of distal enhancers, or the context-dependent effects of TF function (see Discussion).

These findings show that modeling gene expression as a function of TF association at gene promoters can elucidate the functions of specific TFs. To determine a level of confidence in each TF's activating and repressing activity, we performed repeated subsampling of TFs and promoters for training and testing of models. For each TF, we built 500 models based on a random 70%/30% split of promoters and repeated subsampling of 79 other TFs to analyze with the factor of interest. This ensured that a range of estimates for gene expression impact was gathered for each TF. We then identified those TFs with the largest positive and negative impact on gene expression and assigned a significance based on the fraction of submodels in which the TF showed significant impact on gene expression (Fig. 2B). The majority (n = 359) of the TFs we examined showed a positive impact on gene expression, reflecting the more common role of TFs as positive regulators of expression. These also included factors associated with active chromatin such as ASH2L, KMT2A, and KMT2B (Supplemental Table 9). We also identified 26 TFs with a negative impact on gene expression in the models that were significant in at least 50% of all submodels (Supplemental Table 9), indicating their consistent association with a lower level of expression when localized to promoters. These included the confident prediction of a known repressor, REST (also known as NRSF), and several members of the repressive KRAB-ZNF family, including ZNF334, ZFP14, and ZNF140. This is consistent with a model in which these factors localize to regulatory elements to directly or indirectly decrease transcription of nearby genes. Many of the other factors have not been described as transcriptional repressors, including RREB1, ZNF660, and AHR, indicating we have identified novel putative transcriptional repressors. We explored the distribution of peaks for these 26 candidate repressors and compared them with the top 26 candidate activators. We found that, in general, candidate repressors had fewer peaks than candidate activators (Supplemental Fig. 15), consistent with a “hit and run” model of transcriptional repression (Shah et al. 2019). Comparing the distribution of peaks across cCRE types, we found that the majority of peaks for each type were found in promoter-like signature (PLS), proximal enhancer-like signature (pELS), and distal enhancer-like signature (dELS) regions and that distribution of the fraction of peaks in these three region types did not differ significantly between the top candidate activators and candidate repressors (*P* > 0.05, Mann–Whitney *U* test).

We next set out to confirm these predictions with functional data using our lentiMPRA data set. We identified promoter elements in our lentiMPRA data set whose genomic coordinates were bound by at least one of our 26 candidate repressors, the top 26 candidate activators, or a control set of 26 random TFs not found in either set. We compared the promoter regions bound with at least one of these TFs with promoter regions bound by a similar number of TFs but lacking the TF of interest. As the model predicted, regions with one of the 26 top predicted activators had higher activity in the MPRA compared with regions lacking one of these activators (Fig. 2C; Supplemental Table 10). Correspondingly, the activity of regions with a candidate repressive DAP was lower than the activity of regions bound by similar numbers of TFs (Fig. 2C; Supplemental Table 10), except in the case of one to five DAPs bound, which had only 10 observations in this data set, so it is likely a result of noise. These candidate repressor patterns held for most categories of the number of DAPs bound when looking at nonpromoter elements (Supplemental Fig. 16; Supplemental Table 11). We noted that REST was consistently the strongest candidate repressor, with other candidate repressors having a lower fraction of significance across submodels, a smaller repressive effect size, or both (Fig. 2B), consistent with its well-characterized role in repression (Huang et al. 1999; Ballas and Mandel 2005; Ooi and Wood 2007). To confirm that other candidate repressors still showed a repressive effect, we performed additional analysis and found that the general repressive trend of these factors held when removing the effect of REST binding (Supplemental Fig. 17; Supplemental Table 12). Last, the random set showed a pattern inconsistent with either case, with lower expression levels for low numbers of TFs bound and higher expression levels for high numbers of TFs bound (Fig. 2C; Supplemental Table 10). Similar patterns were also found in the Agarwal lentiMPRA elements (Supplemental Figs. 18, 19; Supplemental Tables 13, 14). We also assessed each of our top 26 candidate repressors and activators individually by performing a paired comparison of elements bound or not bound by the factor of interest and matched for the number of factors bound (Supplemental Fig. 20; Supplemental Table 15). We note that findings are generally concordant, with activators having a higher distribution of differences than repressors. We explored the possibility that GC content may confound these findings by comparing GC content differences between matched sequences (Supplemental Fig. 21), and found that the distributions were centered at zero and were balanced in either direction, suggesting that GC content could not explain the differences observed. In some cases, predicted activators did have a lower activity level than did the matched controls, but we note that these cases were almost universally nominally significant (*P* ≤ 0.05) (see asterisk in Supplemental Fig. 20; Supplemental Table 15), with ZNF501, ZFX, ZFY, and ASH2L showing a greater degree of discordance with predictions. This may be a reflection of the fact that the vast majority of factors are predicted to have an activating effect in our models, indicating that replacement with another factor is likely to result in a replacement with a stronger activator. These observations generally confirm our predictions of TF activating and repressing activity.

To further show the utility of our model, we sought to direct binding of these activating and repressing factors to elements using known sequence preferences to activate or repress MPRA activity. We included in our MPRA a set of test elements with known motifs from the JASPAR database for eight putative activating and five putative repressing TFs. These motifs were inserted into two different promoter elements with randomized insertion location, orientation, and spacing (see Methods). We normalized reporter gene expression to control promoters to explore the impact of adding one, two, or five motifs to the promoters, as it has previously been noted that the addition of multiple motifs increases signal (Smith et al. 2013). We found that, of the eight tested candidate activators, five (ATF1, CEBPG, FOSL2, NFYC, and NRF1) show the expected behavior when a single motif is added, whereas three (MYC, ZNF317, and ZNF331) show the opposite effect (Fig. 2D; see *P*-values in Supplemental Table 16). Although this was unexpected, we note that for many TFs there are known context or cobinding dependencies of function, such as the finding of MYC mediating repression when binding with ZBTB17 (Walz et al. 2014). Additionally, we note that for ATF1 a single motif results in activation, whereas additional motifs result in lower signal than with only a single motif added. These results may be because of the artificial nature of the assay; in this case, the insertion of multiple motifs did not lead to a simple interpretation. Of the five tested candidate repressors, all showed the expected negative impact on expression. These general trends remained true when restricting to only one or the other of the two promoter elements (Supplemental Figs. 22, 23; see *P*-values in Supplemental Table 16).

### Analysis of HOT sites shows that ABC score increases with DAP occupancy

HOT sites are regions of the genome with a high number of associated DAPs (Yip et al. 2012), and their biological meaning is an area of ongoing interest (Wreczycka et al. 2019; Hudaiberdiev and Ovcharenko 2023). In particular, several lines of evidence suggest that factor association can occur indirectly (Gordân et al. 2009; Worsley Hunt and Wasserman 2014; Nie et al. 2020), and this may contribute to HOT site formation. Given the large number of ChIP-seq data sets available in HepG2, we sought to provide new insight into the biology and genome organization that produce HOT sites. Although 38.5% of cCREs were bound by a few DAPs, a substantial fraction was bound by large numbers of DAPs (Supplemental Fig. 3; Supplemental Table 3). We defined HOT sites as discrete regions with peaks called in ≥25% of the DAPs assayed, as suggested by our previous study (Ramaker et al. 2020). We identified 13,001 HOT sites in HepG2, representing only 1.7% of all discrete DAP-associated regions but 52% of all DAP peaks across experiments in HepG2 cells. We note that HOT sites occur primarily in promoters or in pELS or dELS elements (Supplemental Fig. 3; Supplemental Table 3); 72.0% are in proximal promoter regions (i.e., overlapping PLS and pELS cCREs), and 27.5% are distal regions (i.e., overlapping dELS). We found that as the number of factors increases, HOT sites become more enriched for promoters (Supplemental Fig. 4; Supplemental Table 3).

Because of this association between HOT sites and promoters, we further explored the relationship between promoter activity and promoter type in these regions. In our MPRA, we observed a general positive correlation between the number of DAPs associated with a promoter and the activity of that promoter element in the MPRA (Fig. 1B; Supplemental Table 4), as has been previously reported (Partridge et al. 2020; Ramaker et al. 2020; Agarwal et al. 2023). Similar to other reports (Chen et al. 2014), we found that HOT promoter regions are primarily in CGIs (6431 of 7411 promoter HOT sites) and frequently at annotated housekeeping genes (1581 of 7411 promoter HOT sites) (Hounkpe et al. 2021) and that the likelihood any given housekeeping gene promoter is a HOT site is very high (94.5%).

To help understand the function of HOT loci and to compare them with other DAP-associated regions in the genome, we performed a principal component analysis (PCA) on a binary matrix of all regions bound by at least three DAPs while noting which DAPs are associated at each region. We confirmed that previously noted associations between PC1 and PC2 (Partridge et al. 2020) are maintained with the number of factors bound and proximal–distal distinctions, respectively (Supplemental Figs. 24, 25). We also noted that ABC scores (Fulco et al. 2019) derived from high-resolution intact Hi-C data for HepG2 showed relationships with both PC1 and PC2 (Supplemental Fig. 26). The strength of connectivity as measured by ABC score increases along PC1 (number of factors bound in a region, Spearman's ρ = 0.2592, *P* ≤ 2.2 × 10^−16^) and decreases along PC2 (proximal vs. distal elements, Spearman's ρ = −0.0724, *P* ≤ 2.2 × 10^−16^), suggesting that there are stronger loop interactions when a large number of DAPs are bound.

### Motif placement in distal–promoter pairs suggests that 3D interactions contribute to HOT site formation

**Figure 3.**
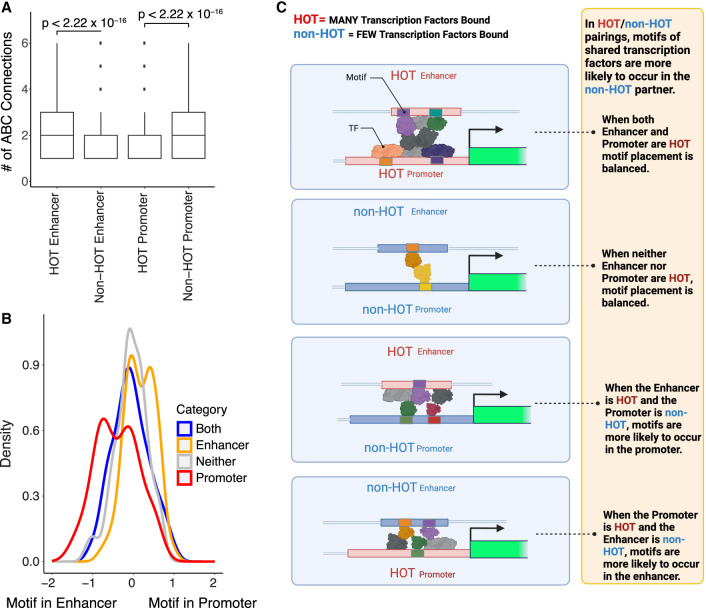
Looping contributes to shared TF binding between putative enhancers (labeled “enhancer” for plot simplicity) and promoters at HOT sites. (*A*) Box plot shows the number of loops (*y*-axis) for putative enhancers and promoters when they are HOT or non-HOT (*x*-axis). Given that at least one loop is present, HOT putative enhancers have more loops than non-HOT putative enhancers, and non-HOT promoters have more loops than HOT promoters (*t*-test *P*-value ≤ 2.2 × 10^−16^). Boxes represent 25%–75% quartiles with line indicating median, whiskers extend to ±1.5 × IQR (interquartile range) past the boxes, and points are observations falling outside of this range. (*B*) Density plot of the natural log of fraction promoters with the relevant motif over fraction of putative enhancers with the relevant motif for loops in which a DAP's peak is found in both the putative enhancer and the promoter. Blue indicates that both the putative enhancer and promoter are HOT. Red indicates that only the promoter is HOT. Orange indicates that only the putative enhancer is HOT. Gray denotes that neither are HOT. Kolmogorov–Smirnov tests were performed to categorize differences between distributions and are presented in Supplemental Table 19. (*C*) Model of motif and TF placement in looping scenarios. The placement of TFs represents hypothetical placement in this model based on the occurrence of motifs and not necessarily an actual example of specific TF binding at specific promoters and putative enhancers based on peak locations observed in data sets.

We next asked whether the concordance in the number of factors bound with ABC score (Spearman's ρ = 0.2591, *P* ≤ 2.2 × 10^−16^) (Supplemental Fig. 27) could highlight distinct DAP binding patterns in promoter-distal interactions at HOT and non-HOT loci. We compared loci in the genome as either HOT or non-HOT, the latter having one or more associated DAP peaks but below the threshold of 25% of the DAPs in our data set. We then observed the number of significant ABC connections that putative enhancers and promoters of each HOT classification (HOT promoter, non-HOT promoter, HOT putative enhancer, non-HOT putative enhancer) had with any other region (Fig. 3A). We observed that HOT putative enhancers have a significantly larger number of connections than do non-HOT putative enhancers and that, conversely, HOT promoters tend to have fewer interactions than do non-HOT promoters. Because, as noted above, 87% of HOT promoters are CGI promoters, which are a mix of ubiquitously expressed and tissue-specific genes, this potentially highlights a mechanism in which DAP regulation is largely proximal for these promoters.

To determine whether connections in 3D space led to peak calls for the same DAPs at both connected regions, we explored the fraction of DAP peak calls shared between a connected putative enhancer and promoter in cases in which one or the other, both, or neither of the regions is a HOT site (Supplemental Fig. 28; Supplemental Table 17). We note that the rate of shared DAP peaks is higher when both sites are HOT and is significantly lower when neither site is HOT (Supplemental Fig. 28, green boxes), consistent with a model in which ChIP-seq peaks might be detected as a result of indirect association.

Given this observation, we wanted to distinguish between two possibilities: The common DAP association is because of (1) independent direct binding of a DAP at multiple sites (either one molecule directly binding the two connected DNA regions simultaneously or two separate molecules binding, one at each region) or (2) direct DAP binding at one locus with an indirect pulldown of nearby connected chromatin in the ChIP-seq data set. To answer this, we identified those DAPs for which a motif is known and quantified percentages of their peaks in promoters or putative enhancers that had a motif present. We found that if both the promoter and putative enhancer are HOT, the motif for the shared DAP is equally likely to be found in either locus of the interacting pair (Fig. 3B; Supplemental Table 18). In contrast, if only one locus is a HOT site, there is a preference for the motif of interest to be found within peaks in the non-HOT site rather than the HOT site (*P* = 3.37 × 10^−7^ Kolmogorov–Smirnov test) (Fig. 3B; Supplemental Table 19). This trend remains true, although reduced in size, when restricting to cases of non-CGI promoters or CGI promoters (Supplemental Figs. 29, 30; Supplemental Table 19). We summarize this model in Figure 3C. For cases in which a peak was observed in both the putative enhancer and the promoter for a given TF and a motif occurs in one of those locations, we hypothesize that a given TF is more likely to be directly interacting with the DNA where its motif occurs, and a peak is found in regions without its motif owing to indirect interactions. These observations are consistent with a model in which the large number of DAPs found bound at HOT sites can often result from indirect association of DAPs at a promoter–enhancer interaction, resulting in an apparent ChIP-seq peak when direct DAP–locus association is unlikely. We note that, among the factors analyzed, MYC and TP53 both contain strong evidence of intrinsically disordered regions based on DisProt annotations (Quaglia et al. 2022). Such disordered regions are thought to be involved in protein–protein interactions (Morris et al. 2021; Chen et al. 2022), and several studies have noted that the disordered regions of these proteins are involved in protein–protein interactions (McEwan et al. 1996; von der Lehr et al. 2003; Fladvad et al. 2005; Di Lello et al. 2006; Wells et al. 2008).

### DAP localization outside of open chromatin regions suggests expansion of candidate CREs

We explored cases of multiple DAPs binding outside of annotated cCREs to assess whether these cases of binding had biological meaning. Early ChIP-seq efforts by ENCODE showed that >94% of DAP peaks occurred in open chromatin regions (Thurman et al. 2012). This is expected because many DAPs prefer binding to nucleosome-free DNA and cooperatively bind with complexes that displace nucleosomes or modify chromatin to make it more open, such as acetylation. The exceptions to this are DAPs associated with heterochromatin or lamin-associated regions, or pioneer factor TFs, which have been found to bind to closed chromatin and lead to nucleosome remodeling and histone modifications that make the region more accessible to additional DAPs (Becker et al. 2017; McCarthy et al. 2021). We expected to observe both of these groups of DAPs in our data set but also could find novel DAP associations with closed chromatin. We found that only 6.3% of DAP peaks occurred outside of any type of cCRE defined as having open chromatin, and 13.4% of peaks occurred outside of an ATAC-seq peak in HepG2. These observations are in agreement with prior analyses (Thurman et al. 2012).

**Figure 4.**
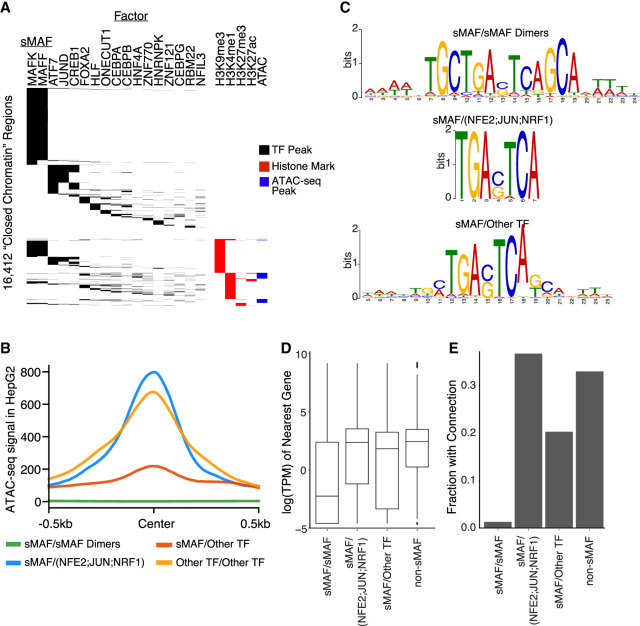
Small MAF (sMAF) proteins bind widely to non-cCRE regions in a sequence-directed manner and show widespread influence on nearby gene expression. (*A*) Heat map of non-cCRE regions with binding of various TF and histone marks. MAFF and MAFK show the largest degree of binding of any such mark. (*B*) ATAC-seq signal (*y*-axis) at regions bound by various TF dimer sets ±500 bp (*x*-axis). sMAF/sMAF (green) dimers show markedly lower ATAC-seq signal compared with other groups, and sMAF heterodimers with known activating cofactors (blue) show high openness. sMAF heterodimers with noncofactors (red) show minimal openness, and heterodimers made of other TFs (orange) show high but broad openness. (*C*) Motifs generated via MEME using regions bound by noted dimer sets. sMAF/sMAF dimers (*top*) show more flanking structure outside of the core MAF motif than do heterodimers of sMAF and non-sMAF proteins. (*D*) Box plot shows the distribution of natural log of expression levels as measured by TPM (*y*-axis) for genes closest to each putative dimer set (*x*-axis). Putative sMAF/sMAF dimers show much lower expression profiles than do other groups. Boxes represent 25%–75% quartiles with line indicating median, whiskers extend to ±1.5 × IQR (interquartile range) past the boxes, and points are observations falling outside of this range. (*E*) Bars show the fraction (*y*-axis) of putative dimer sets (*x*-axis) that have evidence for binding in a looped region, based on Hi-C data. sMAF/sMAF dimers show a significantly lower rate of binding in looped regions than do other dimer types.

For a conservative approach to DAP binding in closed chromatin regions, we limited our analysis to those sites bound by two or more DAPs and regions that were ≥700 bp from a cCRE associated with open chromatin (Supplemental Table 20; Supplemental Fig. 31). We found 16,412 such closed chromatin regions bound by at least two DAPs. These DAP-bound closed chromatin regions had, on average, 2.8 associated DAPs. A majority (95.3%) were outside of an ATAC-seq peak in HepG2, suggesting that most were found in closed chromatin states (Fig. 4A). Analysis of histone modifications at these regions revealed that 70.3% lack any histone signal, whereas another 15.5% of regions had H3K9me3 signal, a mark of heterochromatin and association with the nuclear lamin. This suggests binding by factors outside of open chromatin cCRE regions is more common than expected, accounting for >5% of bound elements in HepG2 cells but occurring in several thousand independent locations far from cCREs. In contrast, a substantial set (12.6%) was associated with H3K4me1, a sign of poised or primed distal elements (Creyghton et al. 2010; Rada-Iglesias et al. 2011). As expected, we found peaks for one or more pioneer TFs at these poised sites, such as FOXA2, ATF7, JUND, CREB1, or CEBP, in 41.8% of the H3K4me1 regions. There are several possible explanations for finding non-cCRE regions bound by several TFs. It is possible these genomic regions do not yet have sufficient evidence to be designated as cCREs but may be designated as such in subsequent versions of the Registry. Some of these regions might represent false positives from DAP ChIP-seq data, although we minimized this possibility by limiting to regions with two or more DAPs bound. We note that although ∼80% of these regions fall in repetitive sequence (Supplemental Fig. 32), there is substantial overlap of other cCRE regions with repetitive sequence content (e.g., 38% of PLS and 57% of dELS overlap with repetitive sequence). We further note that the majority of these regions only show the presence of a few factors, with sMAF factors highly represented, suggesting that false positives caused by repetitive sequence are not a consistent problem across ChIP-seq experiments. We speculate that these regions represent significant DAP genomic localization in closed chromatin. Together, these findings suggest that closed chromatin cCRE regions harbor important regulatory activity and that TF binding should be considered in future efforts to identify novel candidate regulatory elements.

### Binding of small MAF proteins at closed chromatin loci with active or repressive activity

In our analysis of the multiple DAPs bound at closed chromatin regions (Fig. 4A), we found that 44.8% of bound non-cCRE regions showed association with MAFF, MAFK, or, to a lesser extent, MAFG. These three TFs make up a family of small MAF (sMAF) proteins. Unlike the related large MAF family of TFs, the sMAF proteins lack a transcriptional activation domain and are known to act as transcriptional repressors when bound as sMAF/sMAF dimers (Motohashi et al. 2006; Katsuoka and Yamamoto 2016). The vast majority (98.6%) of sMAF coassociations in peaks, putatively indicating sMAF/sMAF dimer binding in these closed chromatin regions, consisted of only MAFF/MAFK, indicating these regions are strong candidates for sMAF repressed *cis*-elements. These regions all fall at least 700 bp away from annotated cCREs and had a mean distance of 2164 bp from the nearest cCRE.

Based on these observations, we explored binding patterns and associated signals for sMAF proteins in our data set. It is known that sMAFs can also heterodimerize with non-sMAF proteins such as NFE2 and act as transcriptional activators (Friling et al. 1990; Rushmore et al. 1991; Katsuoka and Yamamoto 2016). We examined global binding of all sMAF TFs in HepG2 cells and categorized them into two types of loci: (1) bound only by putative sMAF/sMAF dimers, indicated in our data set by peak overlaps for two sMAF TFs, or (2) bound by a putative heterodimer composed of one sMAF with an activating TF, again assumed from peak overlaps. In this analysis, the former group consisted of 30,463 loci and the latter of 1281 loci. The two groups have very distinct characteristics. The sMAF/sMAF dimers are found in regions with little to no ATAC-seq signal, whereas sMAF heterodimers show strong open chromatin (Fig. 4B). The motif found at sMAF/sMAF dimers was the full T-MARE element, with a TGC on each end, whereas the sMAF heterodimers produced a motif of only the core TRE sequence (Fig. 4C). As expected, the expression of the nearest genes to sMAF/sMAF dimers are significantly lower than those nearest to sMAF heterodimers or to a control set of DAP-bound regions (Fig. 4D). We then asked whether the sMAF bound regions show evidence of looping, possibly indicating a mechanism for direct repression of a connected gene. We used ENCODE Hi-C data and found almost no evidence for a loop originating at sMAF/sMAF dimers, whereas sMAF/cofactor bound loci loop as frequently as regions bound by any two random DAPs (Fig. 4E). Thus, sMAF/sMAF dimer binding at closed chromatin appears to have a widespread repressive impact on gene expression through mechanisms other than looping, whereas the heterodimer behaves as an activating TF.

## Discussion

In this study, we generated ChIP-seq data for nearly half of all DNA-associated proteins expressed in HepG2 cells, including representatives of all major families of sequence-specific TFs and spanning the full range from highly expressed to nonabundant and from previously well studied to largely unknown. This substantially larger and more biologically diverse catalog allowed us to seek specific insights into individual TF functions and to evaluate more global patterns of genomic organization and gene regulation. The vast majority of known ENCODE cCREs that are openly accessible in HepG2 cells showed evidence of significant ChIP signal by at least one of our 680 assayed DAPs, and most remaining open cCREs that scored as unbound were computationally predicted to be highly preferred by at least one assayed DAP. We show the power of this data set by (1) identifying candidate *trans*-repressors, (2) exploring binding patterns in distal-promoter interactions, (3) untangling the contributions of such binding to HOT sites, (4) uncovering candidate cCREs that lack open chromatin but are bound by DAPs, and (5) exploring vignettes of a specific class of TFs and their activities.

We found that the majority of cCRE sequences are bound by a DAP that currently has ChIP-seq data available (Fig. 1A; Supplemental Table 2), and explored the likelihood of covering apparently unbound cCREs that would find a peak given ChIPing of additional factors (Supplemental Fig. 6). We found that many of these unbound regions are predicted to have a high affinity for a DAP with existing ChIP-seq data (Supplemental Fig. 7; Supplemental Table 6) and that some regions may be bound but have failed to call a peak owing to false-negative error, based on bigWig signal (Supplemental Fig. 8). Together, these findings suggest that better methods for determining DAP–DNA associations may further elucidate regulatory information in existing data.

We tested functional activities of elements across a wide range of genomic elements with a large lentiMPRA data set and supplemented the resulting data with recently published independent functional data in the HepG2 cell line (Agarwal et al. 2023). Evidence for 26 DAP candidate repressor elements emerged, and this finding was robust to removal of a major canonical repressor, REST. Although the ranges in Figure 2B were large, this is expected as many TFs display context-dependent regulatory behavior. This is highlighted by our discussion of sMAF genes and their differing behavior based on binding partners in Figure 4. Another example of this kind of behavior is in the RFX family, which has a dimerization domain used for homo- and heterodimerization both within the RFX family (Reith et al. 1990; Morotomi-Yano et al. 2002; Aftab et al. 2008; Sugiaman-Trapman et al. 2018) and with other TFs (Caretti et al. 2000). Their interactions have been hypothesized to be important for transcriptional activity (Reith et al. 1994), including activation and possible repression (Iwama et al. 1999; Zhu et al. 2000). Our study highlights the possibility that RFX1 and RFX3 appear to have a repressive effect in HepG2 cells (Fig. 2A). Further study of this family and a deeper examination of TF cooperation and competition will reveal further nuances of individual TF activity. This study, however, has provided broad hypotheses for the function of hundreds of TFs. We also note that the ability to detect repressors, in particular, is complicated by the possibility of hit-and-run repressors (Shah et al. 2019), and thus, a factor with repressive activity may not have been captured by ChIP-seq. The complicated nature of TF behavior outlined above may explain in part the unexpected behavior of some predicted activator motifs in our MPRA seen in Figure 2D, such as MYC, ZNF317, and ZNF331.

The lentiMPRA functional data were also highly useful for supporting analyses derived from the correlative data represented by the ChIP-/CETCh-seq experiments. These data supported the general notion that genomic elements with DAP binding are more likely to be functional and indeed that there is a correlation between the number of bound DAPs and the activity of these elements.

We also found evidence that HOT sites in promoters represent functional binding rather than mere hyper-ChIP-able regions (Wreczycka et al. 2019), as the average element activity continued to increase with genomic binding signal as factors are added, even for highly bound regions. In contrast, the increase in element activity seen with increasingly HOT promoters was not true for highly bound distal elements (Fig. 1B; Supplemental Table 4). A possible explanation for this result is found in the analysis of ABC connections, ChIP-seq peaks, and motif locations explored in Figure 3. We found that, for a given HOT region, when making an active connection with a non-HOT region, specific explanatory motifs are more likely to occur in the non-HOT region. We also found that HOT putative enhancers are more likely to have a higher number of connections than non-HOT putative enhancers (Fig. 3A). It is known that a single enhancer can regulate multiple genes (Fulco et al. 2016; Han et al. 2018), and this finding may help distinguish interesting properties about multitarget versus single-target enhancers. Finally, as the number of factors bound at a putative enhancer increases, the expression of genes with ABC support continues to increase (Fig. 1D; Supplemental Table 8). These observations are consistent with a model in which highly active enhancers regulating multiple promoters have apparent ChIP-seq peaks owing to close 3D association with promoters at which factors are directly bound. In this model, when removed from their larger genomic context, these distal regions would have far fewer factors directly bound than the ChIP-seq data would suggest, leading to a lower overall activity. A key caveat in making element activity correlations with TF occupancy, open chromatin elements, and 3D interactions is that the element sizes and context for these are very different from the MPRA assays. MPRA elements are ∼170 bp, whereas typical conserved distal elements are 450 bp; active promoter regions are often larger than that. In the native chromosome, the entire element is operating and is doing so with the option to interact with other nearby elements. Thus, MPRA assays, with their impressively large data outputs, are a valuable foundation to support future multielement and larger-sized element efforts.

Although Figure 3C highlights one possible model for understanding HOT sites as partially a function of indirect association owing to 3D interactions, we note that there are many other mechanisms that may explain large numbers of factors binding at the same location. These may include the known protein–protein interactions through dimerization domains, such as those known for the sMAF and RFX families, or the noted condensation of factors through the phase transition of their activation domains, as recently noted (Boija et al. 2018).

We found that, for closed chromatin regions bound by any two (or more) factors, sMAF/sMAF dimers are by far the most common binding pair. It has been reported that sMAF factors can bind as repressors or can cobind with other DAPs to activate transcription (Katsuoka and Yamamoto 2016). We here found evidence that sMAF/sMAF dimers play a role in repression through mechanisms that do not appear to involve looping. Whether or not they are bound in known annotated cCRE regions, these dimers occur in closed chromatin, and nearby genes show a much-reduced level of expression compared with genes near sMAF heterodimers with an activator, or other cobound pairs of TFs. These observations may also be related to the previously mentioned hit-and-run mechanism of repression (Shah et al. 2019). If this is the case, then sMAF proteins may be only responsible for maintenance of repressed state, whereas another factor may have induced the repressed state.

These analyses show the power of large ChIP-seq data sets, coupled with functional assays, in a specific cellular context. This includes the ability to mine specific factor behaviors and corresponding *cis*-element classes that are obscured by aggregating binding data across cell and tissue types. This recognizes that many TFs have target sites and activities (e.g., enhancing vs. repressing) that depend on cell type because of isoform coding differences and post-translational activity differences. Furthermore, bound DNA elements are typically compound sequence structures whose integrated functional output is a nonlinear combination of the action of any given bound factor. This means that analyses at the level of element subtypes within the environment of a single cell type give important leverage.

There remain hundreds of sequence-specific TFs (Lambert et al. 2018) for which high-quality ChIP-seq data sets are not yet available. Having such data sets in a context with a large number of other factors for which there is ChIP-seq data will allow for a more refined and concrete understanding of these factors’ functions. Promising tools are being developed to predict the activity of factors (Schreiber et al. 2020; Avsec et al. 2021), yet these tools require high-quality data sets in defined contexts for accurate training. This underlies the need for continued work in generating and analyzing ChIP-seq data sets. The current effort is limited by the fact that the vast majority of ChIP-seq data are in cancer cell line contexts, which affects the identity of factors to be assayed as well as the relevance of these findings to noncancer biology. As methods for ChIP-seq continue to be improved, and as complementary improvements are made in high-throughput *cis*-element assays, we expect a richer and more predictive understanding of the *cis*–*trans* code that controls genome activity.

## Methods

### Data analysis

Data analysis was performed using R versions 3.6.1 and 4.1.0 (R Core Team 2010), as noted in appropriate scripts.

### ChIP-seq data sets

We downloaded processed optimal peak calls of all HepG2 ChIP-seq data sets available on the ENCODE portal on July 6, 2021, and supplemented the data sets with those performed locally at the HudsonAlpha Institute for Biotechnology, which were not uploaded to the ENCODE portal. In-house data were processed using the same ENCODE ChIP-seq processing pipeline to be consistent with data from the ENCODE portal.

### Selection of preferred ChIP-seq data sets

For DAPs that were represented by multiple ChIP-seq data sets, we identified a preferred data set based on the number and severity of audits present on the ENCODE portal. We identified a list of unacceptable audits as follows:

- Failed IDR,
- Low read depth,
- Control low read depth,
- Extremely low read depth,
- Poor library analysis, and
- Severe bottlenecking.

  For this set of audits, in cases of multiple data sets for the same DAP, we identified the number of audits contained in each data set. If one data set contained fewer of these audits than the others, we chose the data set with the smallest number of audits, including zero. If more than one data set had this smallest number of audits, we compared the audits within each data set to determine whether a given data set had a less severe set of audits (e.g., low read depth vs. extremely low read depth) or a smaller number of audits overall. Ranking of the severity of audits was in the order of the list above, from most severe to least severe. Finally, if data sets were not distinguishable, we chose the data set with the largest number of peaks. In cases in which a data set was available on the ENCODE portal, such a data set was always preferred to those not from the portal.

The same process was used to identify K562 ChIP-seq data sets for comparison in binding-expression models.

### Gene expression data

We used RNA-seq data sets from the ENCODE portal under accession ENCSR181ZGR for HepG2 and ENCSR885DVH for K562. We took the count table of the two replicates and took the average TPM as the TPM for a given transcript. For genes with multiple transcription start sites (TSSs), we identified the transcript with the highest TPM for the purposes of selecting an isoform.

### cCRE catalog

The V4 cCRE human data set was downloaded from the ENCODE portal under accession ENCSR800VNX.

### ATAC-seq data sets

ATAC seq peaks were acquired from the ENCODE portal under accession ENCSR291GJU on February 5, 2021.

### HOT sites

Using only those DAP data sets (excluding histone marks) that were preferred (see above), we restricted peaks to the central 100 bp. We merged all peaks and determined the number of DAPs contributing to each merged region. We then identified HOT sites as those that had at least 25% of all factors bound. As we had 680 unique DAPs in our data set, HOT sites are defined as locations with 170 or more unique DAPs bound.

### gkm-SVM model construction and use

HOT sites and the ENCODE exclusion list were removed from peaks, and peaks were then sorted by −log_10_ *P*-value. Up to the top 10,000 peaks were then extracted. Null sequences were generated using the nullseq_generate script from gkm-SVM with the parameters -G -x 2 -m 1000 -r 1 (Ghandi et al. 2016). We ran gkmtrain to produce a model of sequence preference with -l 8 -k 5 -d 2. We ran this for all data sets. Once made, we applied these models to the regions of the cCRE catalog to which no DAP ChIP-seq data set had evidence of association using gkmpredict.

### Activity by contact predictions

HepG2 intact Hi-C data set ENCSR888DEJ was used to produce ABC predictions and was provided to us by Jesse Engreitz.

### Hi-C data loop calls

The Hi-C loops for the non-cCRE sMAF analyses in Figure 4 were called with the Juicer pipeline (Durand et al. 2016). Resolution calls of 500 bp were used.

### Motif derivation

For each data set, we removed all peaks found in HOT sites or the ENCODE exclusion list, sorted the remaining peaks by −log_10_ *q*-value, and determined the number of peaks remaining. If there were at least 1000 peaks remaining, we then restricted peak width to the ±50 bp surrounding the peak center. Using the top 500 peaks of the data set, we used MEME version 5.1.0's meme-chip method to derive motifs using the following arguments: -dna -meme-mod zoops -meme-nmotifs 5 -meme-minw 6 -meme-maxw 50 -spomao-skip -fimo-skip. We then performed several tests to identify those motifs that were sufficiently enriched in our data. Namely,

1. The motif's *e*-value as determined by MEME must be ≤0.05.
2. We identified the top 501–1000 peaks in the data set, extracted the sequence ±150 bp from the center of each peak, and determined the number of times the motif is observed based on FIMO (Grant et al. 2011). We then performed the same method on null-matched sequences, sampling many times to determine a mean and standard deviation of the number of times a motif is observed. We took the *z*-score of the observed number of motifs in the test set and calculated a *P*-value based on the standard normal distribution. If the *P*-value was ≤0.00001, the motif passed this test.
3. For all peaks past the first 500, using the sequence ±150 bp, we determined the number of times the motif was observed based on FIMO. For those same peaks, we also extracted an additional set of control sequences 150 bp upstream of and 150 bp downstream from the test regions and determined the number of times the motif was observed based on FIMO. If the number of test observations was ≥1.25 times the number of control observation and the motif was found in at least 10% of test sequences, the motif passed this test.
4. Finally, we determined whether the *e*-value of the motif found in meme-chip's centrimo tests was ≤0.05.

If the motif passed tests 1, 2, and either 3 or 4, then the motif was considered real for the data sets in question.

### ABC–HOT relationships with regard to motif placement

Using ABC predictions, we determined, for each putative enhancer and promoter region, whether the putative enhancer, promoter, both, or neither overlapped with a HOT site. For each category (both HOT, promoter HOT, enhancer HOT, neither HOT), we then determined, for each factor with a derived motif (see above), which distal-promoter interactions had a peak for that factor in both the putative enhancer and the promoter. For each of those cases, we then determined whether that factor's motif was found within the TF's peak in the putative enhancer or promoter. We identified the fraction of cases in which the TF was in the putative enhancer and the fraction in the promoter and then determined the ratio by taking the log(fraction_promoter/fraction_enhancer). We then created a distribution plot of these ratios for each of the four categories of HOT interactions. Kolmogorov–Smirnov tests were used to identify significant differences among the distributions. When considering all promoters, we restricted to cases of factors that had at least 100 instances of shared peaks (i.e., the peak for the TF was found in both the putative enhancer and the promoter of a pair) to avoid concerns of noise overly influencing log-ratio distributions. For restrictions to only CGI and non-CGI promoters, there were too few TFs with 100 shared peak observations to perform statistical tests, so we used all available TF data.

### CGI and non-CGI promoters

CGI annotations in promoters were taken from Illingworth et al. (2010), and coordinates were converted from hg19 to hg38 using the command-line version of the liftOver tool of the UCSC Genome Browser (Hinrichs et al. 2006).

### Binding-expression models

We identified TSSs for genes based on RefSeq TSSs acquired April 12, 2019. For genes with multiple TSSs, we identified the transcript with the highest expression level (see above) and used that TSS for the gene. We took the ±500 bp upstream of the gene's TSS and determined which TFs showed association with the promoter based on overlap with peaks of the gene. We then constructed a linear model by training on 70% of genes and testing on 30% of genes, with 10× cross-validation to determine the ability of the linear model to predict gene expression.

Once this was determined, we then wanted to investigate the estimated impact of each TF on gene expression. We first subsampled different numbers of TFs to predict expression levels, taking different sample sizes of TFs 500 times, building a model based on 70% of TSS, and testing on the remaining 30%. We found that little improvement was to be found for sample sizes of more than 80 TFs (Supplemental Fig. 33). Therefore, for every TF, we performed 500 samples, subsampling the factor and an additional 79 factors, and built a model of gene expression based upon factor binding. For each subsampled model, we determined the estimate of the factor of interest and whether or not it was found to be significant in that model. This showed the average ability of a factor to determine expression, independent of the other factors included in the model.

### MPRA data and processing

Processed MPRA data were acquired from Agarwal et al. (2023) and are available from ENCODE (https://www.encodeproject.org/) under accessions ENCSR632EPR and ENCSR463IRX. To determine which factors were considered bound to the corresponding genomic context, we identified the summit of each peak and expanded to ±25 bp in each direction. Those peaks that were fully contained by the hg38 genomic coordinates of these elements were considered bound to the genomic region of the element.

Lentiviral MPRA data were produced by following the protocol of Gordon et al. (2020), with minor modifications. Oligos (n = 138,420) (Supplemental Table 21) were synthesized by Twist Bioscience and were composed of a 170-nt test element with a 15-nt 5′ flanking sequence and a 15-nt 3′ flanking sequence (Supplemental Table 21). First-round amplification of the oligo library was performed as described with 5BC-AG-f01 and 5BC-AG-r01, but a separate first-round amplification was performed in parallel with custom primers 5BC-AG-f01B (5′-CTCACTCAGCCTGCATTTCTGCCAGGGCCCGCTCTAGACCTGCAGGTCGGTTCACGCAATG-3′) and 5BC-AG-r01B (5′-GCTTTCGCTTAGCGATGTGTTCACTTTGCACAGTACCGGATTGCCAAGCTGGAAGTCGAGCTTCCATTATATACCCTCTAGTGAGGACCGGATCAACT-3′). These two reactions gave one library with test elements in forward orientation and one library with test elements in reverse orientation. These libraries were combined in equal quantities before second-round PCR amplification. For barcode association sequencing, we modified the read 1 and read 2 sequencing primers for our dual orientation library to (read 1) 5′-CTGCATTTCTGCCAGGGCCCGCTCTAGACCTGCAGG-3′ and (read 2) 5′-TGGAAGTCGAGCTTCCATTATATACCCTCTAGTG-3′. All other primers and protocol steps followed the published method. We produced lentivirus in 293FT cells using established protocols and filtered media (0.45 μM) to obtain virus. We seeded HepG2 cells in six-well plates (1 million cells per well), and 24 h postseeding spinfected virus into HepG2 cells using established protocols (2000 RPM, 1.5 h). After 48 h of incubation, we extracted RNA and DNA from cells using a aNorgen total RNA purification kit and Qiagen DNeasy blood & tissue kit, respectively; we purified three replicates, using eight wells per replicate (16 RNA columns per replicate). All sequencing runs were performed on NextSeq high-output flow cells. We obtained 151,069,132 reads for barcode association, 454,643,305 total reads for the three RNA libraries, and 167,815,184 total reads for the three DNA libraries (Supplemental Table 22). Signal was calculated by determining the normalized number of reads corresponding to each element in the RNA and the DNA libraries. We observed a mean of 91.74 barcodes per test element. We confirmed that there was a strong concordance among replicates (Supplemental Fig. 34) and that there was sufficient barcode representation for elements (Supplemental Fig. 35). Promoter elements were identified by overlapping with GENCODE human promoter annotations (Frankish et al. 2021). For binding-expression analyses, we used two sequences, H_046 and ENH_HMM_B_1, and inserted either one, two, or five instances of established motifs for a given factor taken from the JASPAR database (Castro-Mondragon et al. 2022). Location and orientation of inserted motifs were randomized. In the case of multiple motif insertions, minimum spacing was also randomized but was always >5 bp.

### PCA analyses

For exploration of the relationship between PCAs of a binding matrix and various genomic features, the protocol was followed as previously described (Partridge et al. 2020). Briefly, we constructed a matrix of DAPs bound across 2-kb genomic bins, restricted to bins with at least three DAPs bound, and performed PCA on the resulting matrix using the princomp() function in R.

### Non-cCRE DAP dimer identification

To identify putative dimers in non-cCRE regions, we restricted peaks of all factors to the summit of the peak ±50 bp. We then identified all cases of two factor peaks overlapping by at least 50 bp. Putative sMAF/sMAF dimers were identified by first finding all such overlaps and then removing any instances of similar overlap with any other factor. sMAF cofactors were identified as listed by Katsuoka and Yamamoto (2016), and putative sMAF/cofactor dimers were identified by first removing all cases of putative sMAF/sMAF dimers and then identifying all cases in which a sMAF peak had at least a 50-bp overlap with a cofactor peak. Finally, putative sMAF/other and other/other dimers were identified by first removing all cases of putative sMAF/sMAF and sMAF/cofactor dimers and then identifying all cases of 50-bp overlap between a sMAF peak and other factor or other/other peaks, respectively.

ATAC-seq profiles over putative dimer regions were generated using deepTools (Ramírez et al. 2016). The heat map in Figure 4A was generated using the ComplexHeatmap package (Gu et al. 2016).

### Repetitive sequence identification

Repetitive sequences were acquired from http://genome.ucsc.edu/cgi-bin/hgTables by selecting the “repeats” group, designating GRCh38/hg38, and selecting the output format as “BED” and saving the resulting BED file.

## Data access

All raw and processed MPRA and ChIP-seq sequencing data generated in this study have been submitted to the NCBI Gene Expression Omnibus (GEO; https://www.ncbi.nlm.nih.gov/geo/) under accession numbers GSE235360 and GSE235477, respectively. Code is provided as Supplemental Code, and both code and relevant data for the creation of plots are available at GitHub (https://github.com/bmoyers/Moyers_et_al_2023_HepG2_TF/).

## Supplementary Material

Supplement 1

Supplement 2

Supplement 3
