# Supplementary material for: Characterization of human transcription factor function and patterns of gene regulation in HepG2 cells
Source: Genome Res. 2023 Nov;33(11):1879–92. doi: 10.1101/gr.278205.123 (PMC10760452; doi:10.1101/gr.278205.123)
Supplement: Supplement 2 [file Supplemental_Figures.pdf]

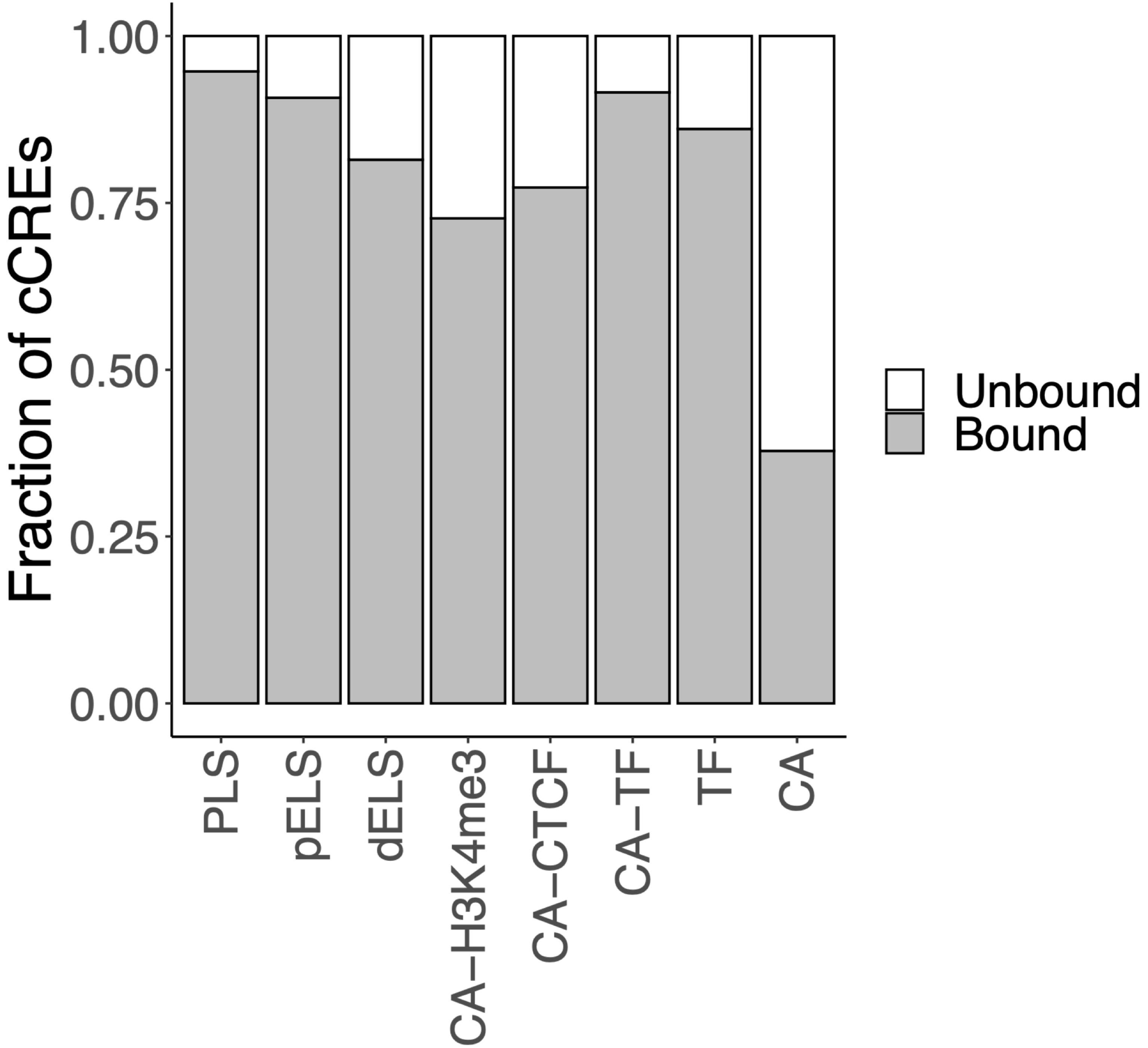

Supplemental Figure 1. Fraction (y-axis) of each cCRE class (x-axis) with at least one DAP associated ("bound") ad those with none in our dataset ("unbound") when restricted to those overlapping with an ATAC-seq peak in HepG2.

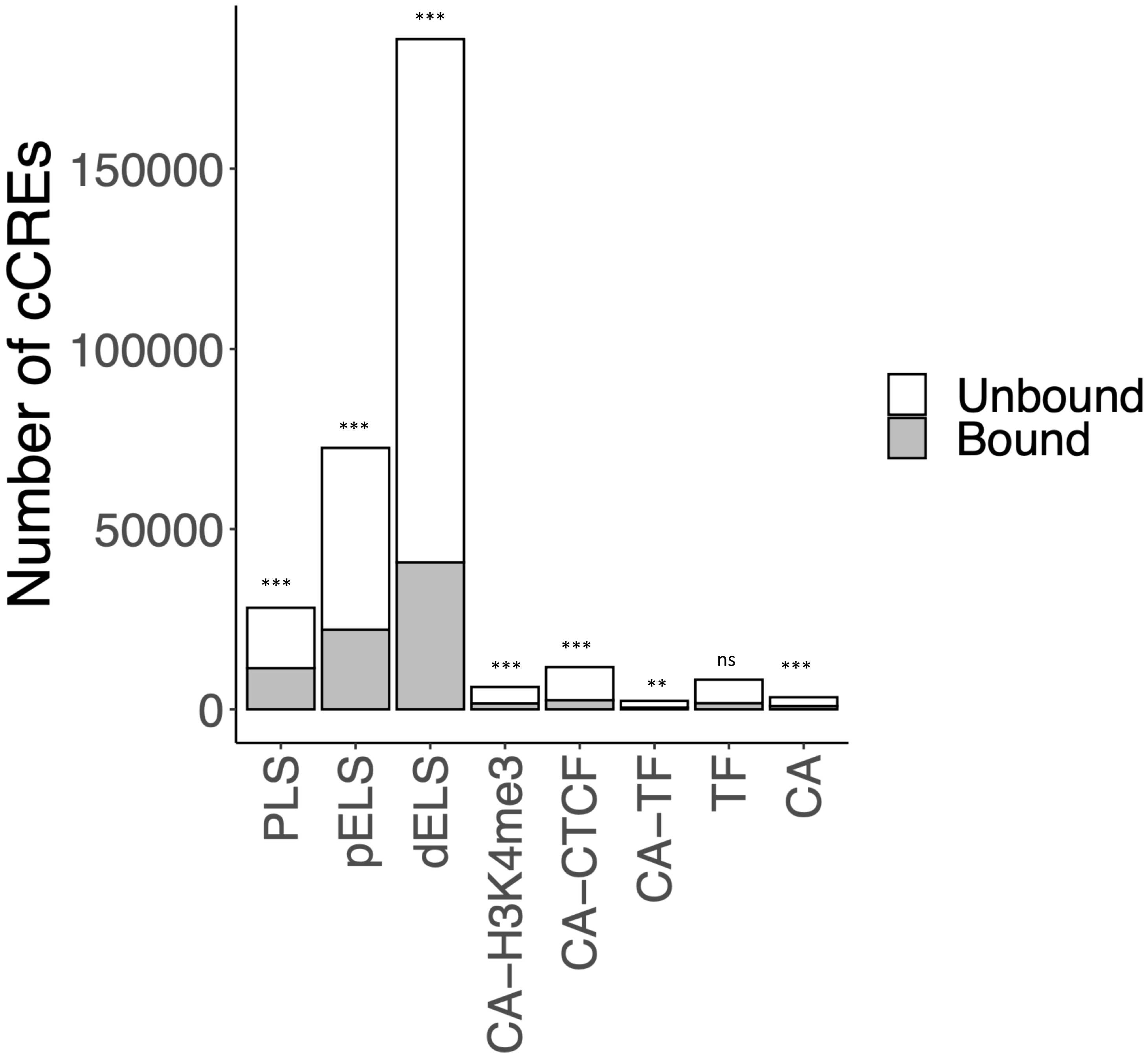

Supplemental Figure 2. Dinucleotide-matched control regions show reduced incidence of TF binding. Control regions for each open chromatin region of a given cCRE were generated using the nullseq_generate.py function of the LS-GKM suite. Overlap of TF binding was then determined, as for Figure 1A. Bars show the number of sites of each control group (x-axis) with at least one DAP association ("bound") and those with none in out dataset ("unbound"). Asterisks show significance of a chi-squared test comparing observed versus control sequences for bound and unbound sets.

*** p<=2.2E-16
** p<=1E-8
* p<=0.05

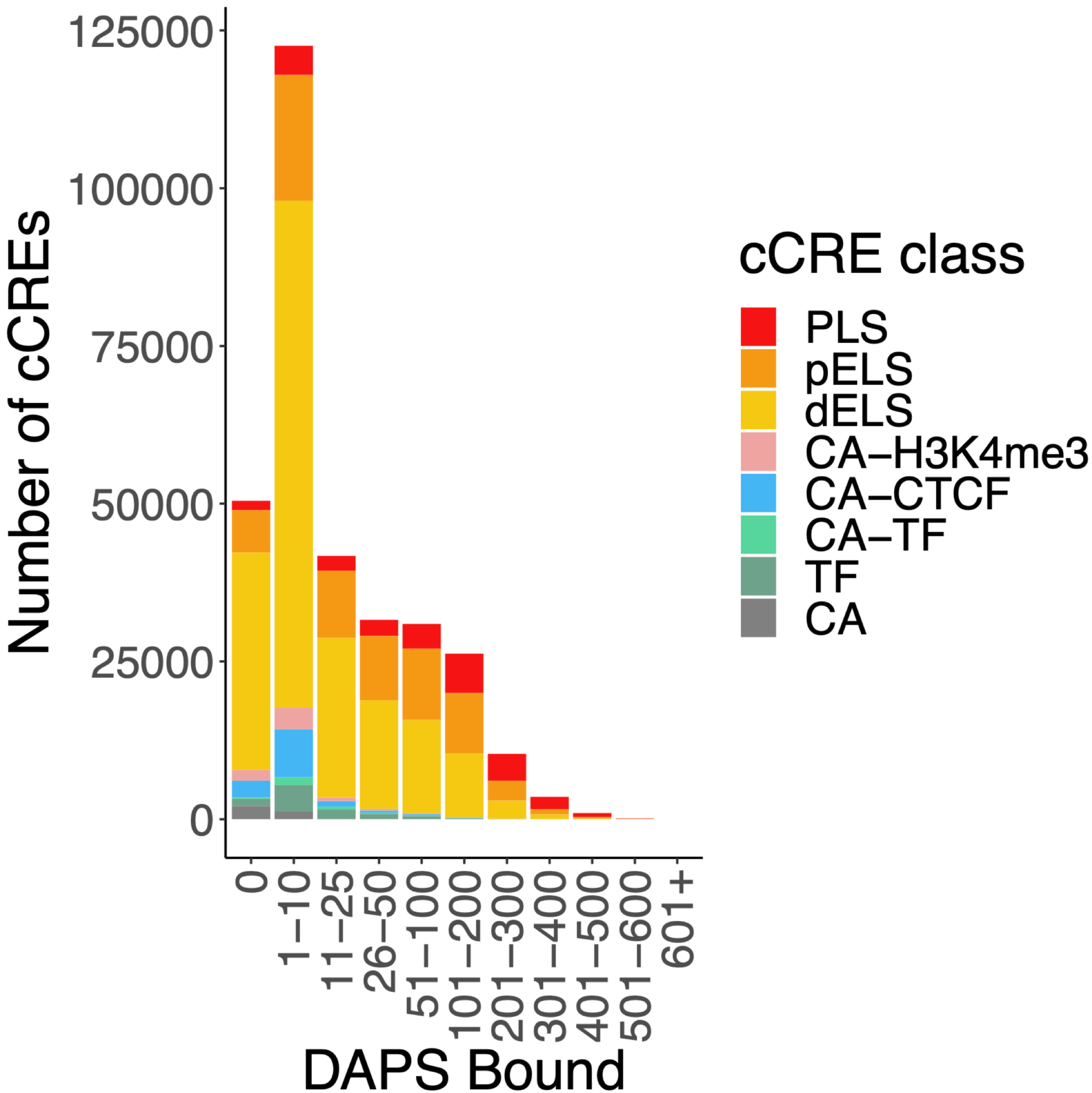

Supplemental Figure 3. Barplot showing the number of regions of each cCRE class bound (y-axis) as a function of binned number of DAPs bound to a region (x-axis). cCRE classes are denoted by color. PLS (red), pELS (orange), dELS (yellow), CA-H3K4me3 (pink), CA-CTCF (blue), CA-TF (light green), TF (dark green), CA (grey). HOT sites are bound by >=170 of our DAPs, and thus represent a portion of the 101-200 bin, as well as all higher bins.

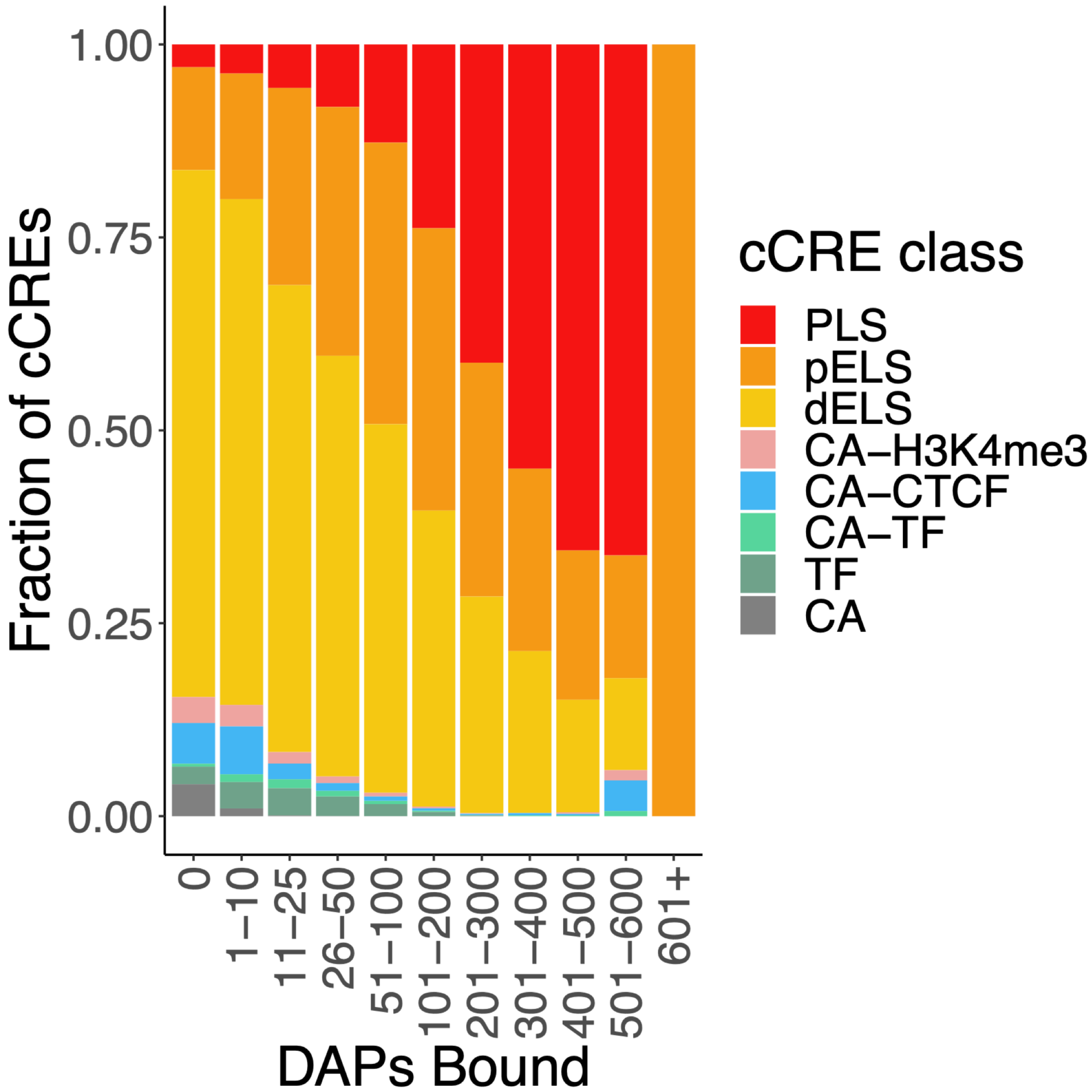

Supplemental Figure 4. Barplot showing the fraction of regions of each cCRE class bound (y-axis) as a function of binned number of DAPs bound to a region (x-axis). cCRE classes are denoted by color. PLS (red), pELS (orange), dELS (yellow), CA-H3K4me3 (pink), CA-CTCF (blue), CA-TF (light green), TF (dark green), CA (grey). HOT sites are bound by >=170 of our DAPs, and thus represent a portion of the 101-200 bin, as well as all higher bins.

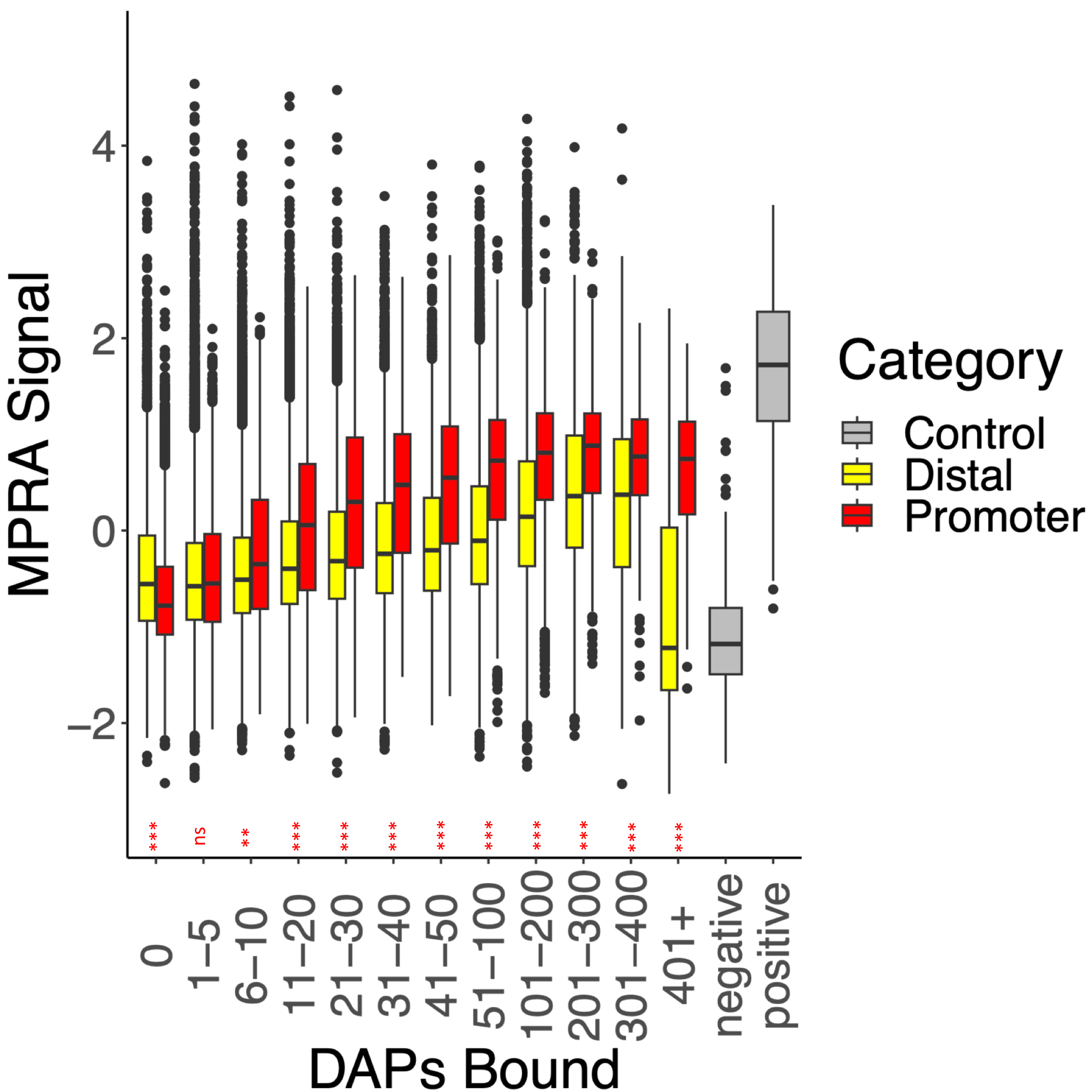

Supplemental Figure 5. Boxplot shows lentiMPRA signal as denoted in Agarwal et al 2023 (y-axis) as a function of binned number of DAPs bound (x-axis) in the genomic region for promoter (red) and distal (yellow) regions, with control sequences (grey) for comparison. Boxes represent 25-75% quartiles with line indicating median, whiskers extend to +/-1.5*IQR (inter-quartile range) past the boxes, and points are observations falling outside of this range. Asterisks denote p-values comparing distal to promoters in each category.

*** p<=2.2E-16

** p<=1E-8

* p<=0.05

When comparing to negative control, all sets are significant at p<=2.2E-16 except for the promoter group at DAPs 0 (p<=1E-8) and Distal at DAPs 401+ (ns)

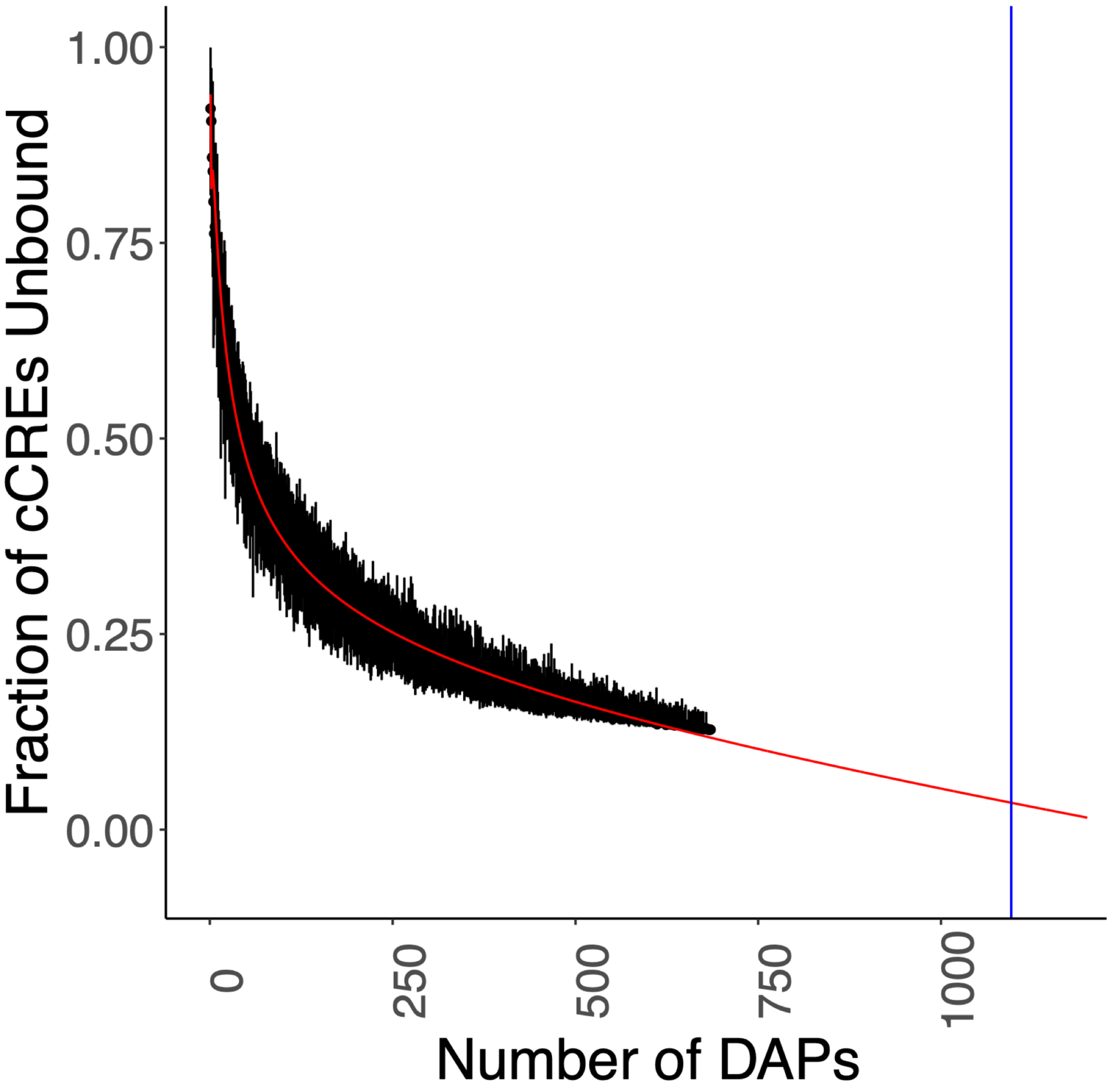

Supplemental Figure 6. When subsampling TFs, there are diminishing returns on the fraction of cCREs covered by at least one experiment when additional DAPs are added. Each subsampling of varying number of DAPs (x-axis) is 20 iterations, with y-axis showing the 95% confidence interval of fraction of total human cCREs not bound in each set of iterations. Red line represents a predictive trend line fit to the model:

$$FractionUnbound \sim \frac{1}{\#DAPs} + \frac{1}{sqrt(\#DAPs)} + \frac{1}{\#DAPs^2}$$

Blue line marks 1096 DAPs, the number if our current dataset were combined with all currently unassayed DAPs with >2 TPM expression in HepG2.

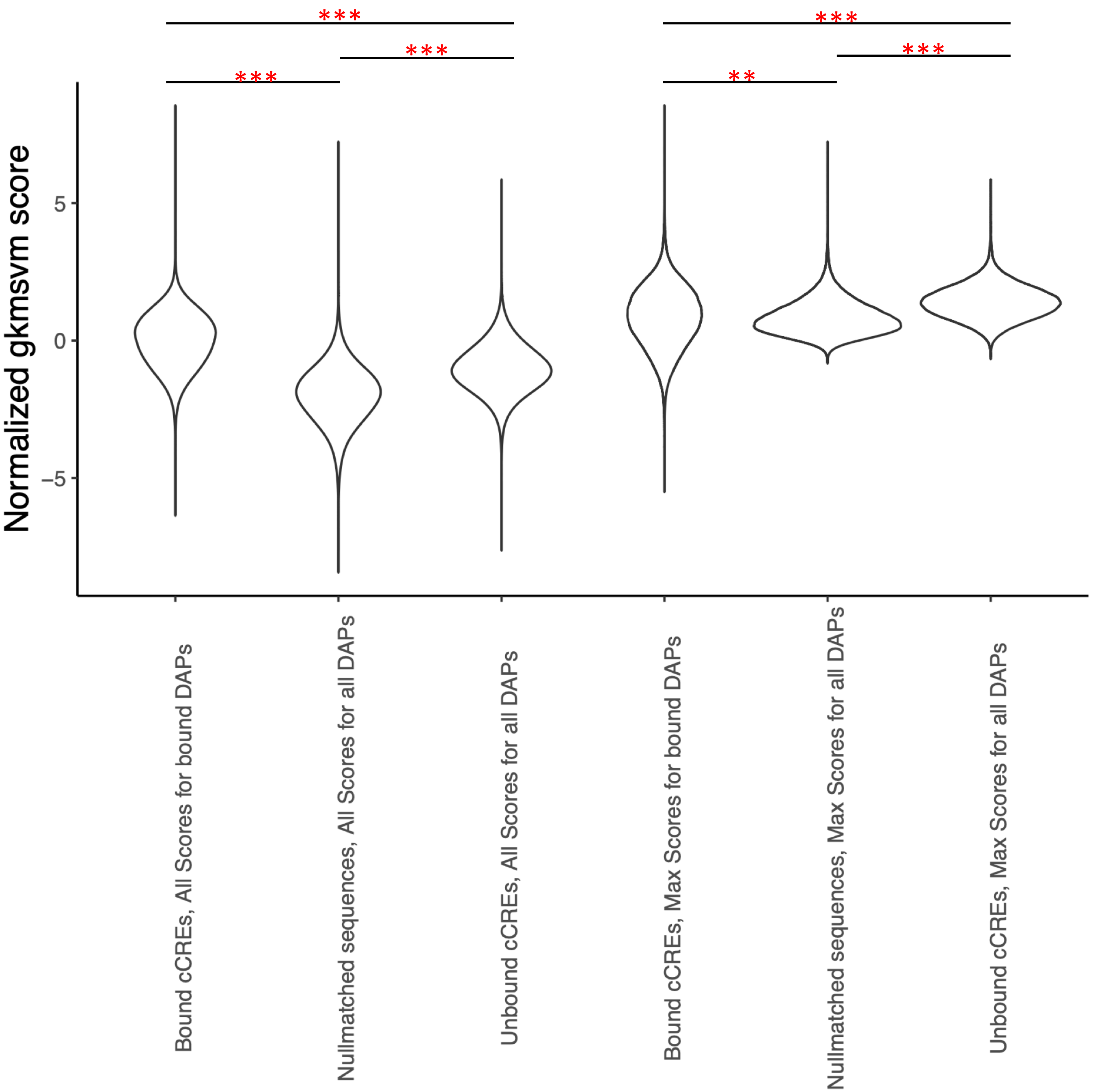

Supplemental Figure 7. gkm predict scores for various genomic regions. We generated dinucleotide matched control sequences for all of the bound cCREs using the nullseq_generate.py scrip from the LS-GKM suite.

”Bound cCREs, All Scores for bound DAPs” shows all normalized gkmsvm scores for all factors bound to each bound cCRE.

“Nullmatched sequences, All Scores for all DAPs” shows all normalized gkmsvm scores calculated across all DAPs for each of the null matched sequences.

“Unbound cCREs, All Scores for all DAPs” shows all normalized gkmsvm scores calculated across all DAPs for each of the unbound cCREs.

“Bound cCREs, Max Scores for bound DAPs” shows the maximum normalized gkmsvm score for a given bound cCRE among all factors bound at that cCRE.

“Nullmatched sequences, Max Scores for all DAPs” shows the maximum normalized gkmsvm score for a given null matched sequence across the scores calculated for all DAPs for that null matched sequence.

“Unbound cCREs, Max Scores for all DAPs” shows the maximum normalized gkmsvm score for a given unbound cCRE across the scores calculated for all DAPs for that unbound cCRE.

The figure shows that, for null matched sequences against the bound cCREs, mean maximum score is lower than the mean maximum observed score of bound cCREs (column 2 versus column 4), and that the mean unbound cCRE maximum score is higher than each of the others (column 6 versus columns 2 and 4). This implies that many unbound cCREs are likely to be bound by at least one DAP, and may represent false negatives in the peak calling pipeline.

** $p \le 2E-8$

*** $p \le 2.2E-16$

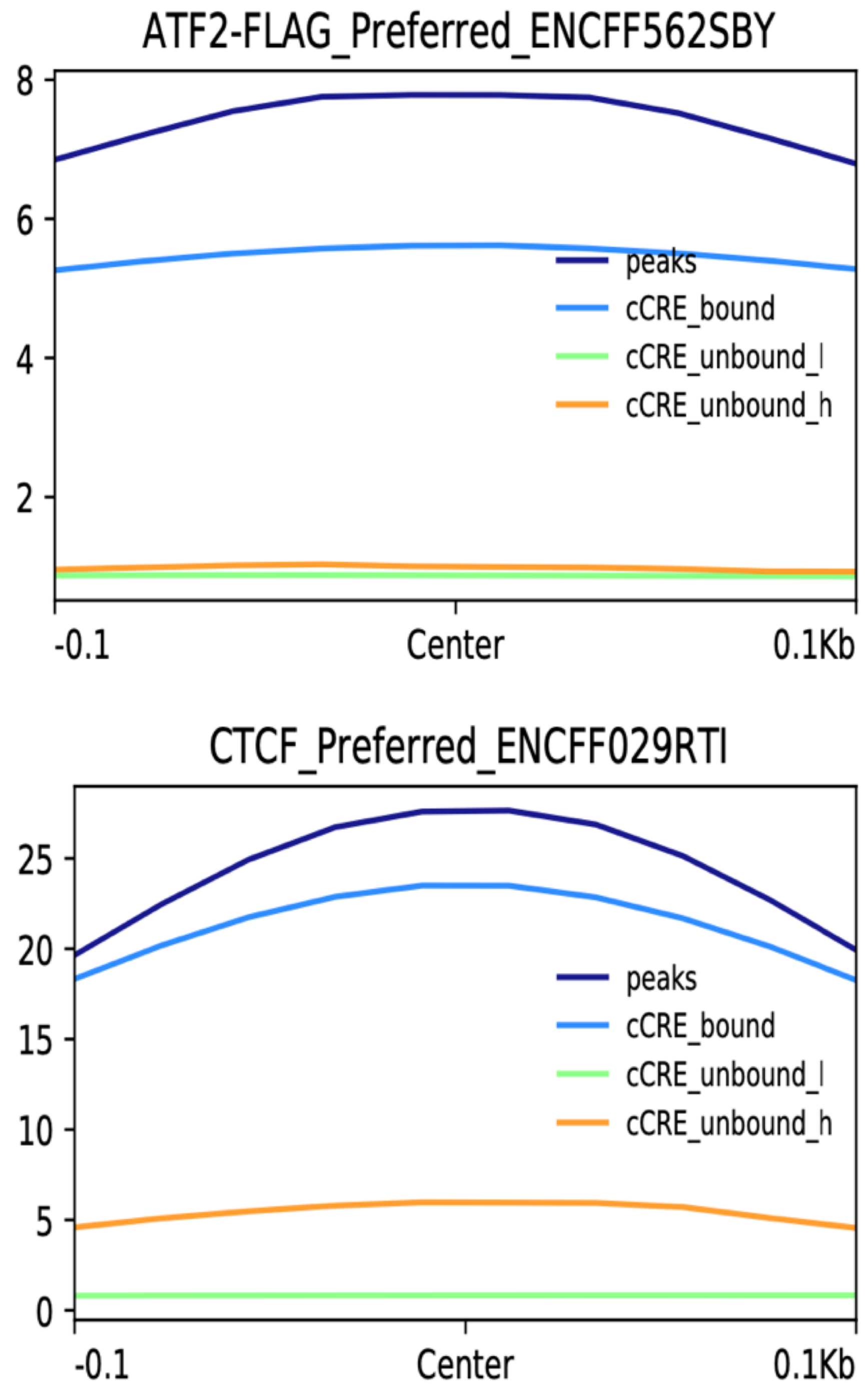

Supplemental Figure 8. bigwig signal summaries produced by deepTools for ChIP-seq experiments for ATF2 (upper) and CTCF (lower) over the following regions:

peaks: Peaks for the experiment in question.

cCRE_bound: the full cCRE sequence for cCRE sequences which overlap with a peak for the factor.

cCRE_unbound_l: the full cCRE sequence for cCREs with no TF peak, and with a gkm-svm at or below the 10th percentile of gkm-svm scores for this TF.

cCRE_unbound_h: the full cCRE sequence for cCREs with no TF peak, and with a gkm-svm at or above the 90th percentile of gkm-svm scores for this TF.

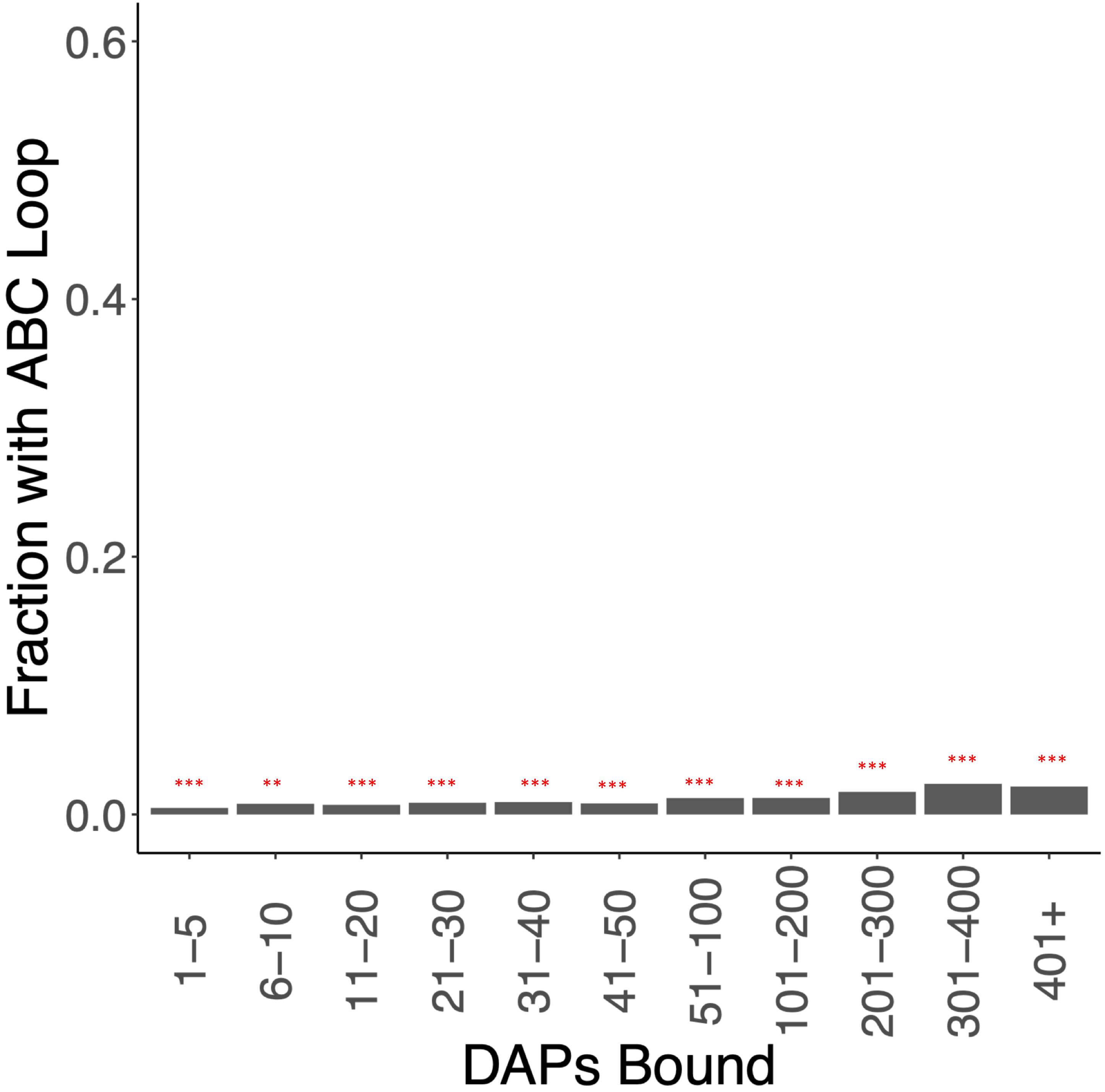

Supplemental Figure 9. Dinucleotide-matched control regions show reduced incidence of ABC looping in most cases. Control regions for each sequences bound by a given number range of DAPs were generated using the nullseq_generate.py function of the LS-GKM suite. Overlap of ABC support was then determined, as for Figure 1C. Graph shows the fraction of control loci with an ABC connection as a function of the binned number of DAPs from which the control element was generated.

*** p<=2.2E-16
** p<=1E-8
* p<=0.05

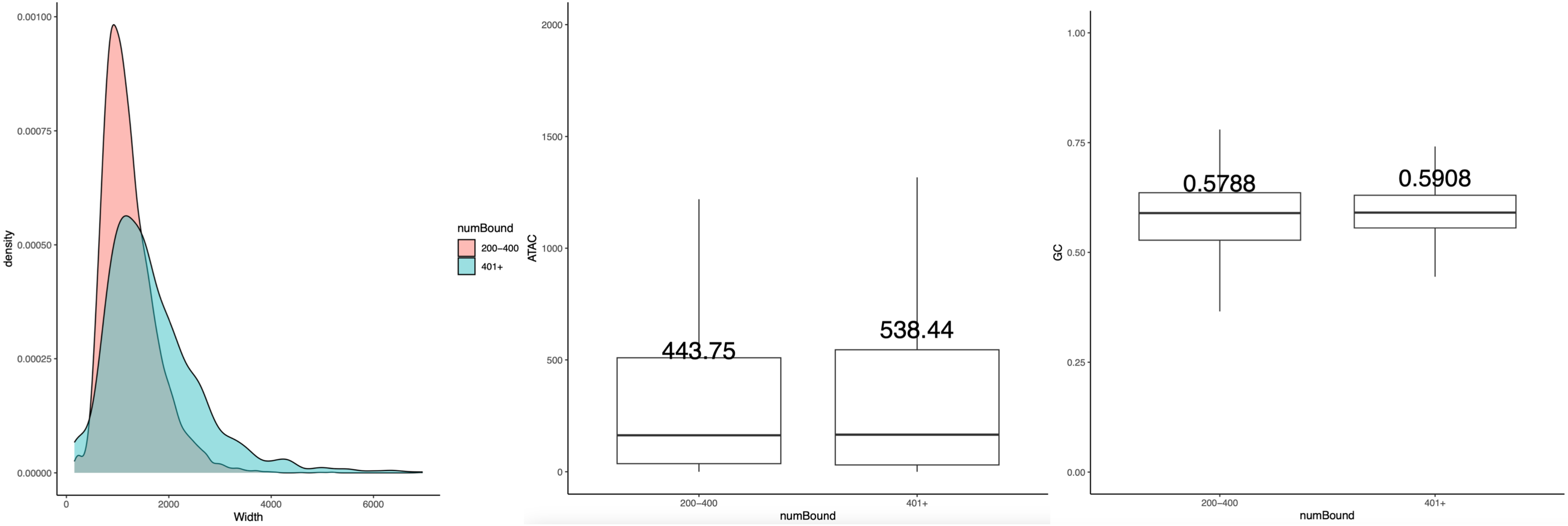

Supplemental Figure 10. Exploration of regions with high numbers of DAPs bound. Left: Size distribution of regions with either 200-400 DAPs bound or 401+ DAPs bound. Center: Comparison of mean ATAC-seq bigWig signal in regions with either 200-400 DAPs bound or 401+ DAPs bound ($p<=2.2 \times 10^{-16}$, Mann-Whitney *U*-test). Right: Comparison of GC content in regions with either 200-400 DAPs bound or 401+ DAPs bound ($p=8.65 \times 10^{-4}$, Mann-Whitney *U*-test)

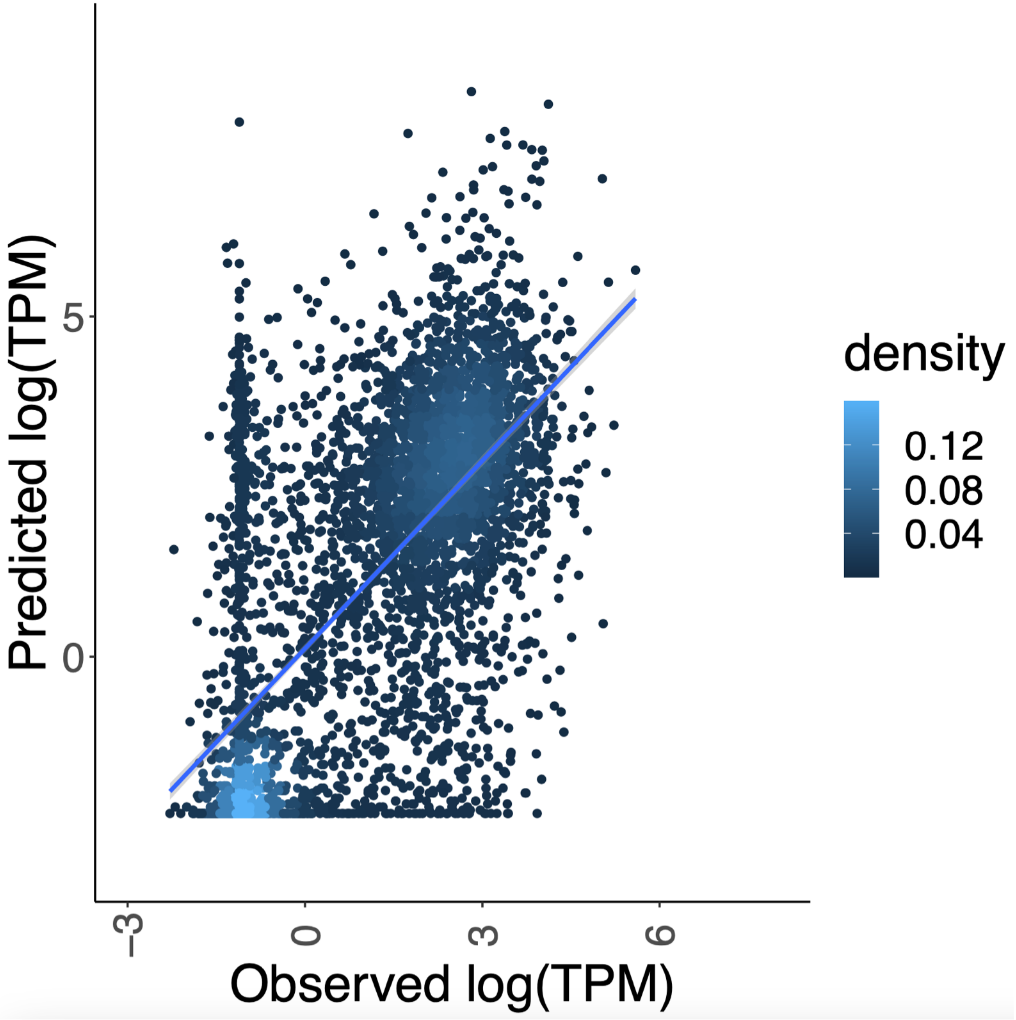

Supplemental Figure 11. Observed (x-axis) versus predicted (y-axis) natural log of gene expression as measured by transcripts per million. A linear model was constructed based on binding of TFs at a gene's TSS +/- 500 bp. Training and testing were performed on a 70/30 split of all genes containing a CpG island. Pearson's correlation=0.65, p<=2.2E-16. Blue line was generated from geom_smooth in the ggplot2 package.

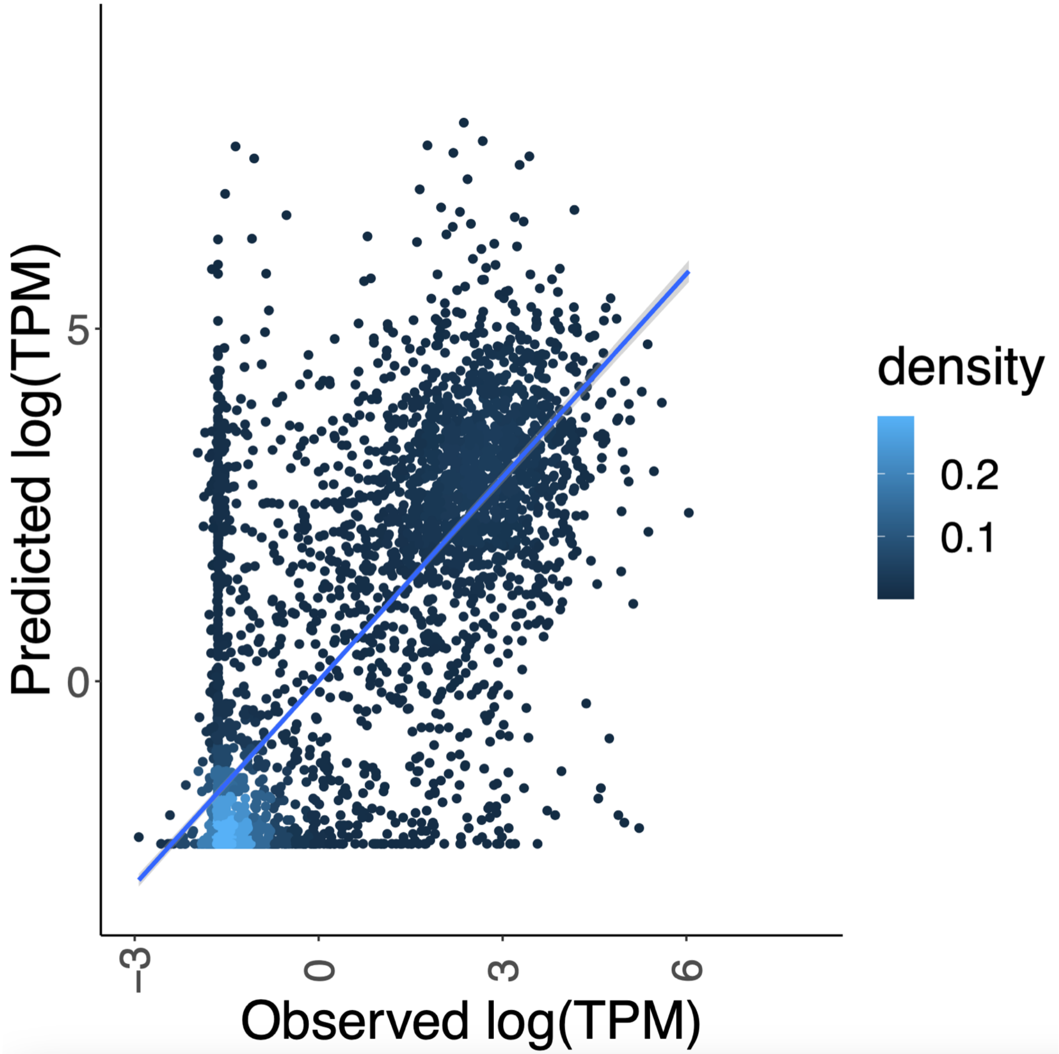

Supplemental Figure 12. Observed (x-axis) versus predicted (y-axis) natural log of gene expression as measured by transcripts per million. A linear model was constructed based on binding of TFs at a gene's TSS +/- 500 bp. Training and testing were performed on a 70/30 split of all genes lacking a CpG island. Pearson's correlation=0.76, p<=2.2E-16. Blue line was generated from geom_smooth in the ggplot2 package.

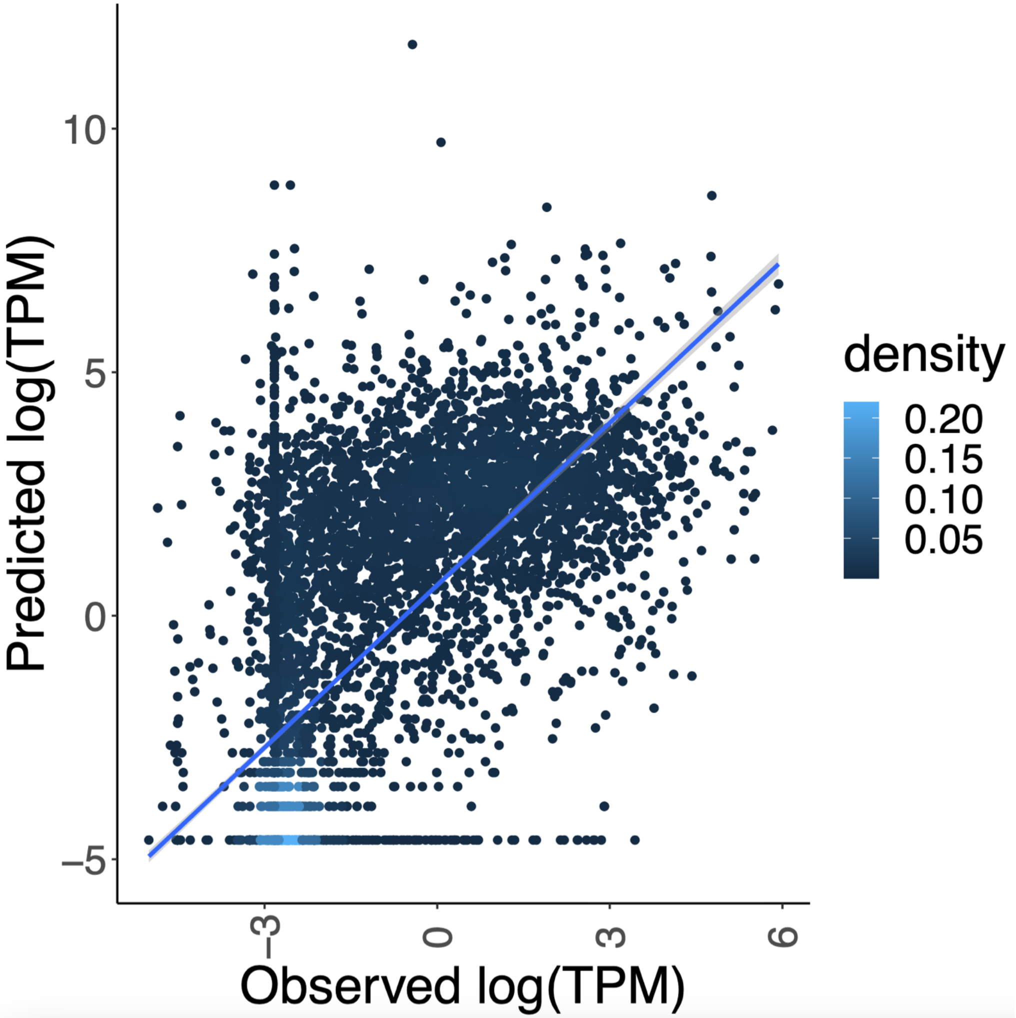

Supplemental Figure 13. Observed (x-axis) versus predicted (y-axis) natural log of gene expression as measured by transcripts per million. A linear model was constructed based on binding of 184 TFs with ChIP-seq datasets available in both HepG2 and K562 cells. Binding at a gene's TSS +/- 500 bp was determined for each cell type. Training was performed using observed binding and expression on 70% of genes in HepG2 cells, and testing was performed using the other 30% of genes in K562 cells. Pearson's correlation=0.67, p<=2.2E-16. Blue line was generated from geom_smooth in the ggplot2 package.

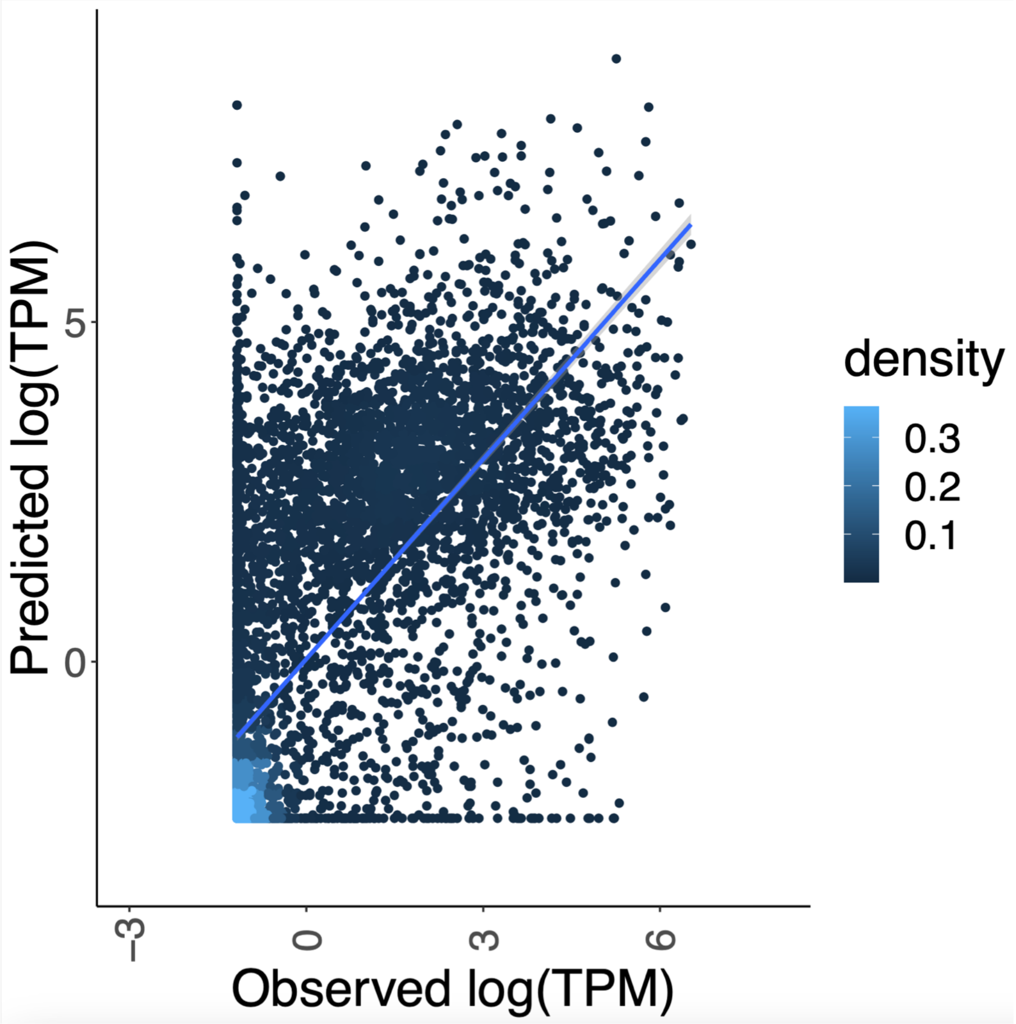

Supplemental Figure 14. Observed (x-axis) versus predicted (y-axis) natural log of gene expression as measured by transcripts per million. A linear model was constructed based on the number of TFs bound at a gene's TSS +/- 500 bp. Training and testing were performed on a 70/30 split of all genes. Pearson's correlation=0.69, p<=2.2E-16. Blue line was generated from geom_smooth in the ggplot2 package.

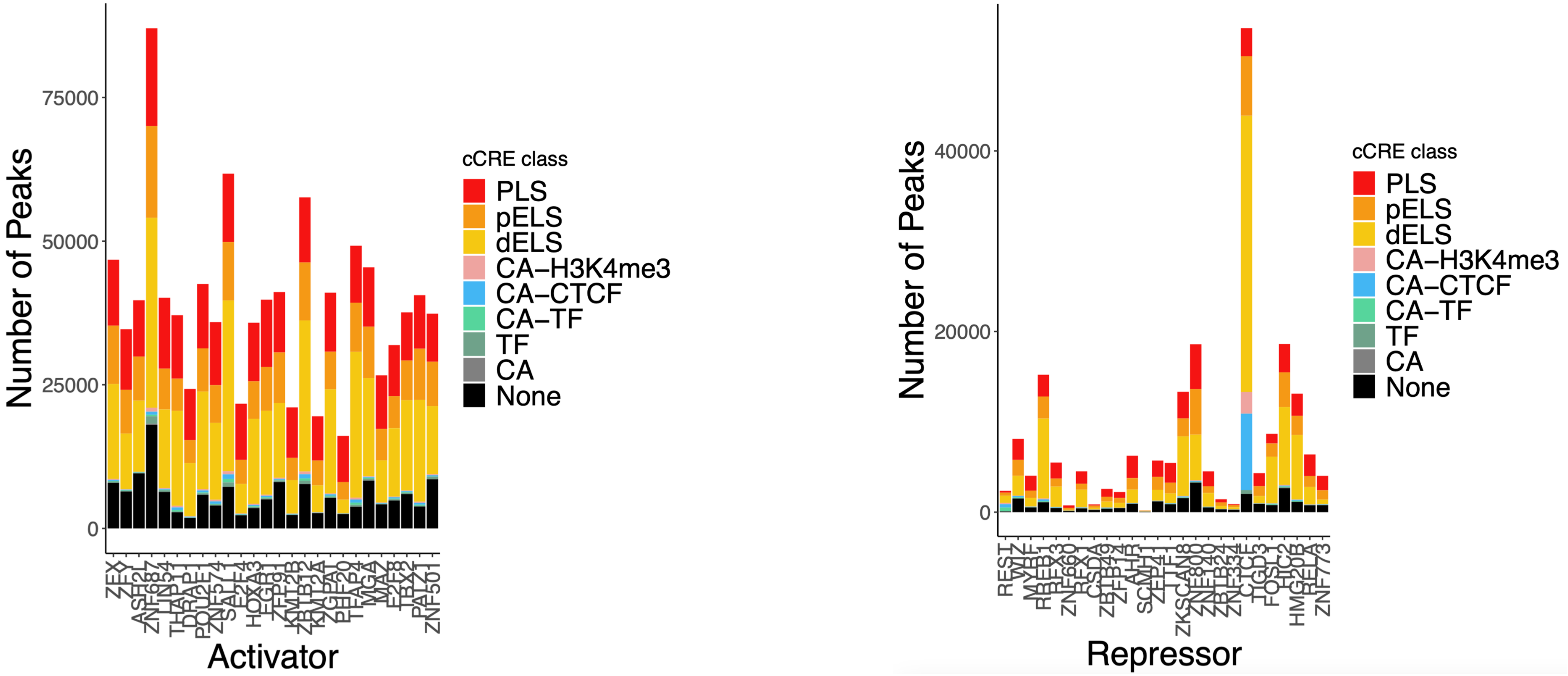

Supplemental Figure 15. Barplots showing cCRE overlap distribution for the top 26 Candidate activators and top 26 Candidate repressors. Plots show the number of peaks overlapping with a given cCRE class (y-axis) for each of the candidates (x-axis). **Left:** Candidate activators. **Right:** Candidate repressors.

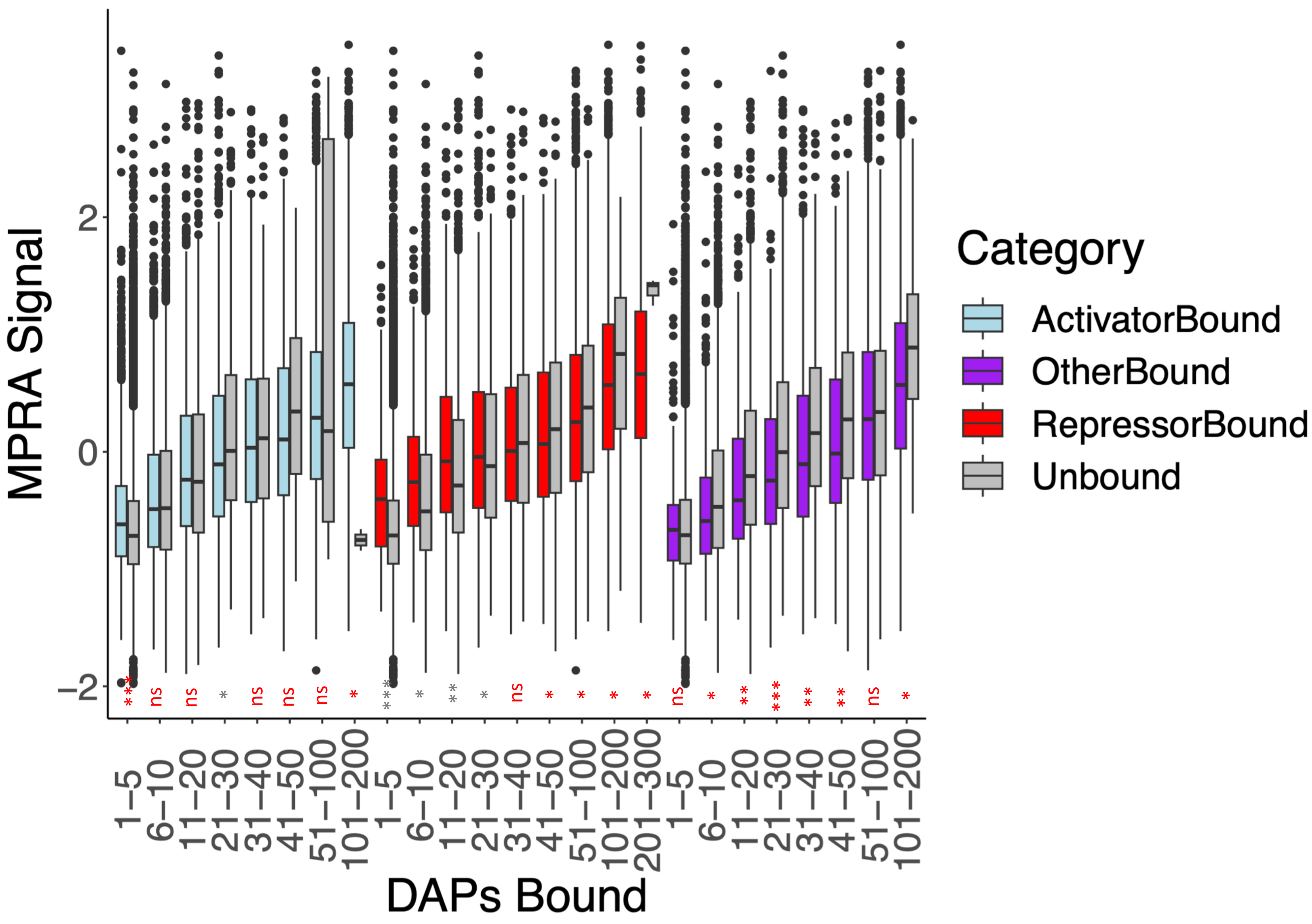

Supplemental Figure 16. Signal in lentiMPRA (natural log of normalized RNA reads over normalized DNA reads) (y-axis) as a function of binned number of DAPs (x-axis) for distal regions either bound by one of the top factors identified in the linear model as an Activator (blue), Repressor (red), or randomly-selected TF (purple), compared to regions which were not bound by one of those TFs for each group (grey), demonstrating activating, repressing, and uncertain activity for each respective group of TFs. Boxes represent 25-75% quartiles with line indicating median, whiskers extend to +/-1.5*IQR (inter-quartile range) past the boxes, and points are observations falling outside of this range. Asterisks denote p-values comparing Bound to Unbound in each category.

*** p<=2.2E-16

** p<=1E-8

* p<=0.05

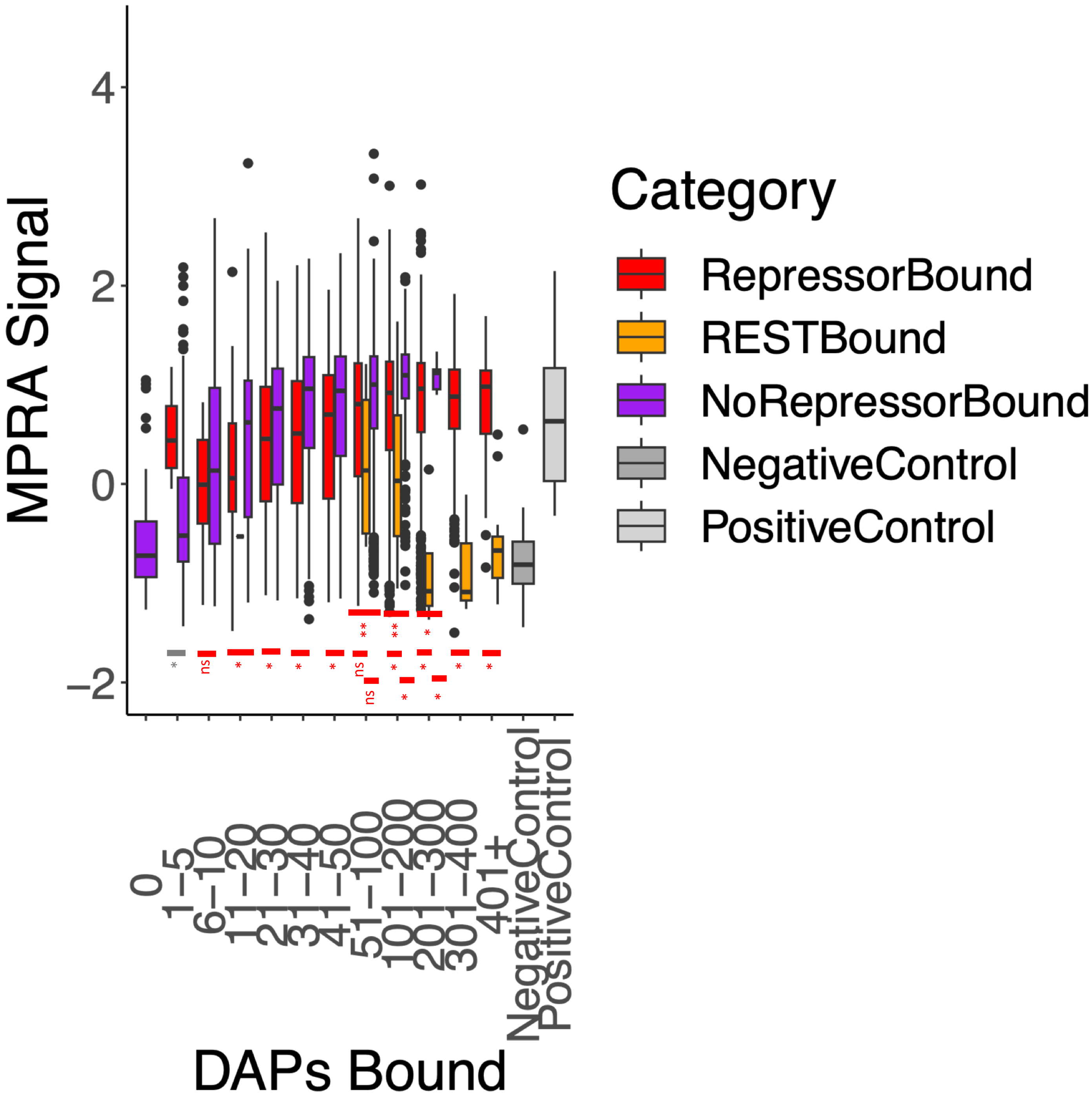

Supplemental Figure 17. Signal in lentiMPRA (natural log of normalized RNA reads over normalized DNA reads) (y-axis) as a function of binned number of DAPs (x-axis) for promoter regions bound by one of the top factors identified in the linear model as a Repressor excluding REST (red), bound by REST (orange), not bound by one of these repressors (purple), compared to regions which were not bound by one of those TFs for each group (grey), demonstrating activating, repressing, and uncertain activity for each respective group of TFs. Boxes represent 25-75% quartiles with line indicating median, whiskers extend to +/-1.5*IQR (inter-quartile range) past the boxes, and points are observations falling outside of this range. Asterisks denote p-values comparing the groups which bars connect.

*** p<=2.2E-16

** p<=1E-8

* p<=0.05

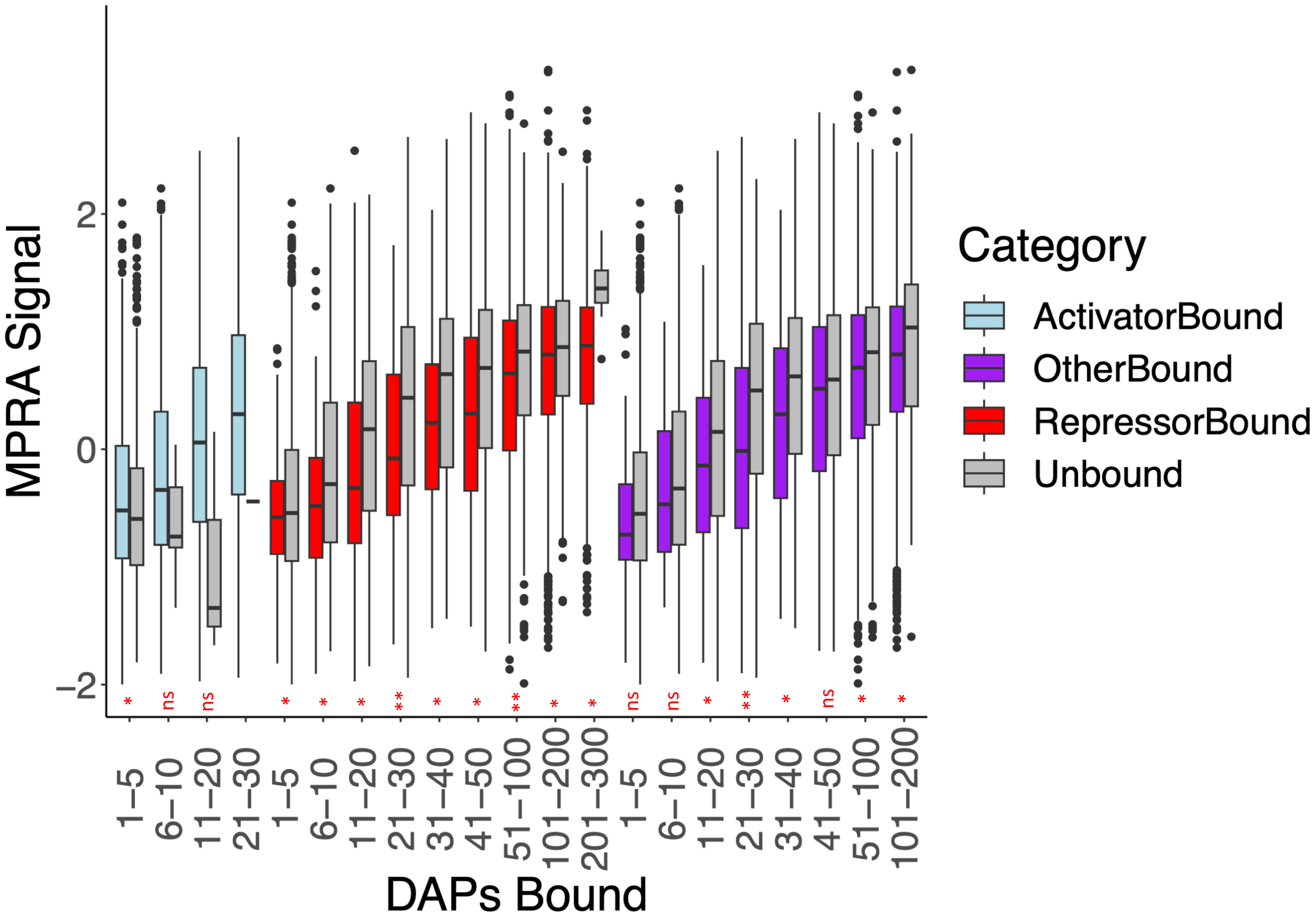

Supplemental Figure 18. lentiMPRA signal as denoted in Agarwal et al 2023 (y-axis) as a function of binned number of DAPs (x-axis) for promoter regions either bound by one of the top factors identified in the linear model as an Activator (blue), Repressor (red), or randomly-selected TF (purple), compared to regions which were not bound by one of those TFs for each group (grey), demonstrating activating, repressing, and uncertain activity for each respective group of TFs. Boxes represent 25-75% quartiles with line indicating median, whiskers extend to +/-1.5*IQR (inter-quartile range) past the boxes, and points are observations falling outside of this range. Asterisks denote p-values comparing Bound to Unbound in each category.

*** p<=2.2E-16
** p<=1E-8
* p<=0.05

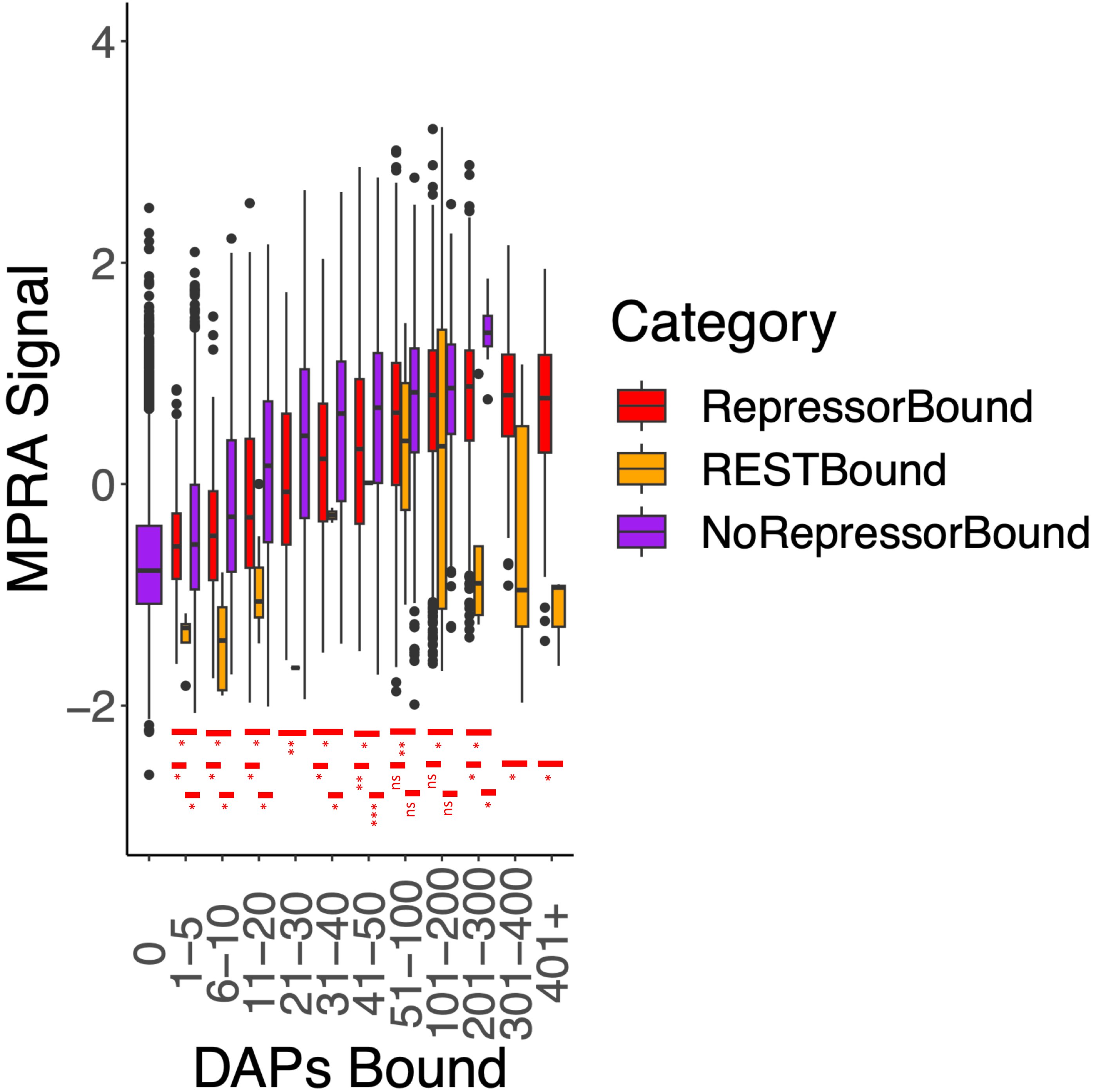

Supplemental Figure 19. lentiMPRA signal as denoted in Agarwal et al 2023 (y-axis) as a function of binned number of DAPs (x-axis) for promoter regions bound by one of the top factors identified in the linear model as a Repressor excluding REST (red), bound by REST (orange), not bound by one of these repressors (purple), compared to regions which were not bound by one of those TFs for each group (grey), demonstrating activating, repressing, and uncertain activity for each respective group of TFs. Boxes represent 25-75% quartiles with line indicating median, whiskers extend to +/-1.5*IQR (inter-quartile range) past the boxes, and points are observations falling outside of this range. Asterisks denote p-values comparing the groups which bars connect.

*** p<=2.2E-16

** p<=1E-8

* p<=0.05

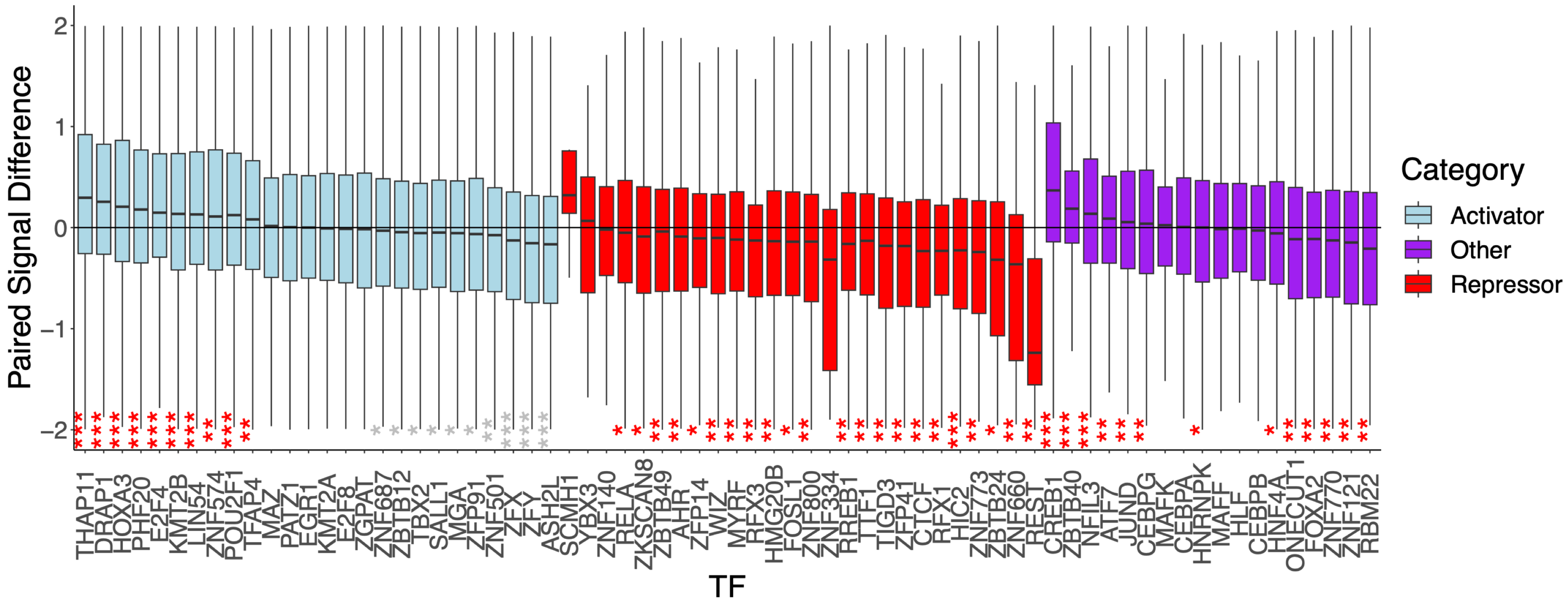

Supplemental Figure 20. Differences in MPRA signal (natural log of normalized RNA reads over normalized DNA reads) (y-axis) for elements bound by a given TF (x-axis) and a comparable element bound by the same number of factors, but missing the TF in question. Candidate Activators are colored in blue, Candidate Repressors in red, and a selection of other TFs in grey. Paired t-tests were used to identify significant differences in means between bound and compared sequences. * = 0.05, **=0.0001, ***<=2.2E-16. Red asterisks represent concordance with expectations, while grey asterisks represent discordance with expectations. Boxes represent 25-75% quartiles with line indicating median, whiskers extend to +/-1.5*IQR (inter-quartile range) past the boxes, and points are observations falling outside of this range.

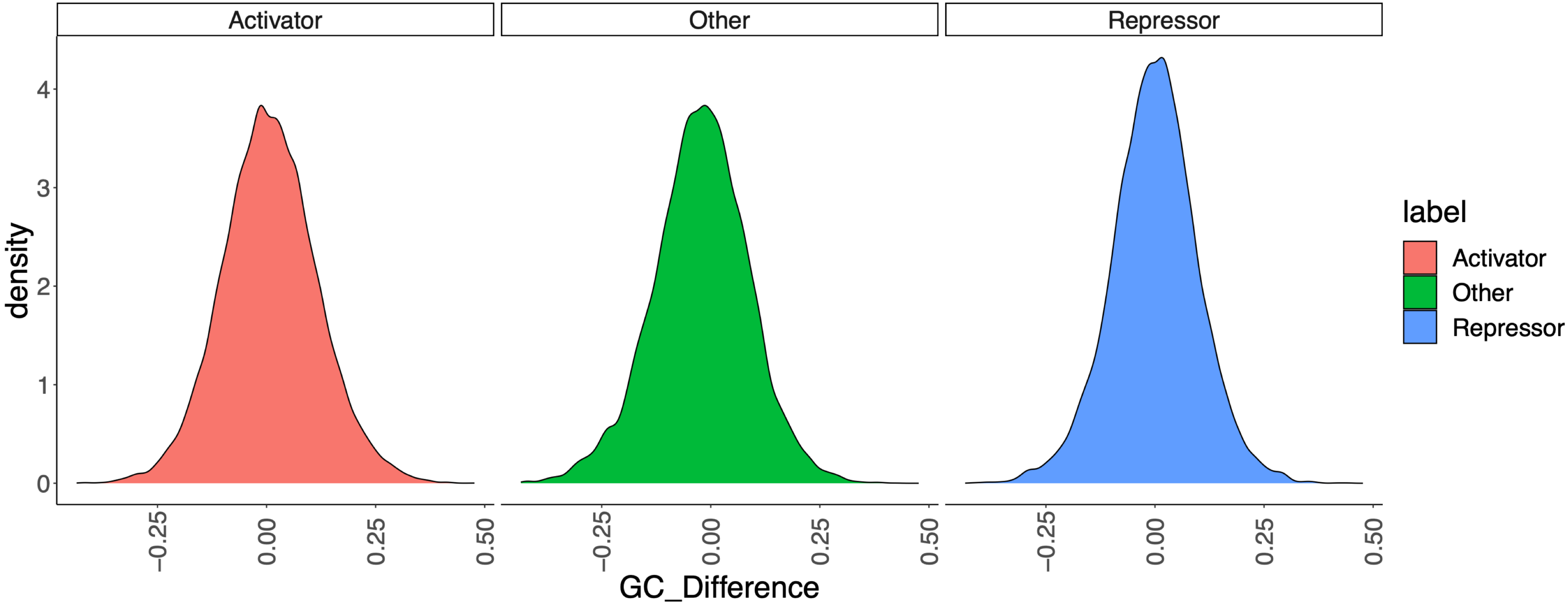

Supplemental Figure 21. GC content difference between matched elements used for Supplemental Figure 18. We find that, for all cases, GC content difference appears approximately balanced with a mean and mode close to 0. This suggests that GC content is not a major contributor to difference in element signal.

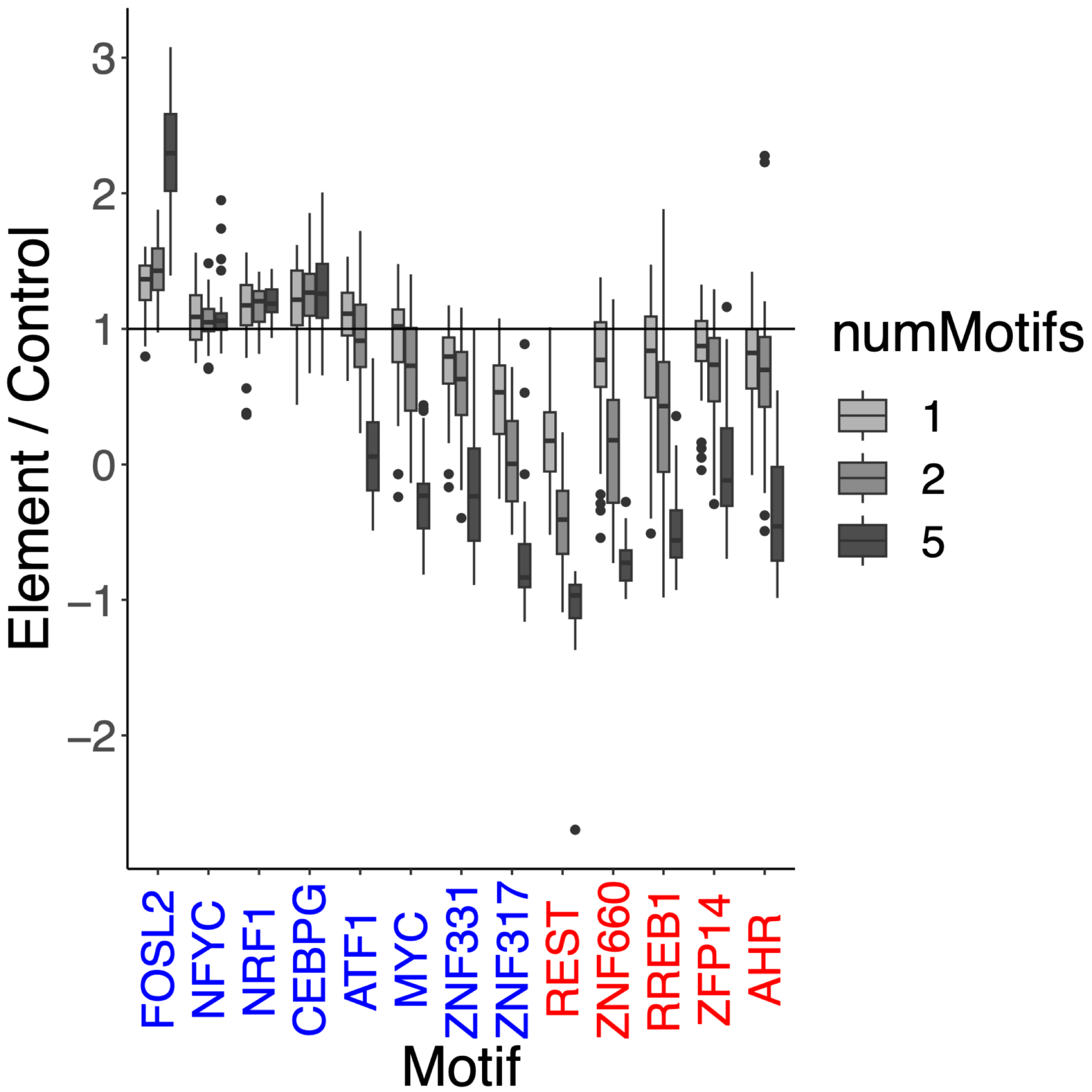

Supplemental Figure 22. Signal in lentiMPRA (natural log of normalized RNA reads over normalized DNA reads) ratio over H_046 control element signal distribution (y-axis) for motifs inserted into enhancer sequences at various intervals (x-axis). A group of candidate activators (x-axis, blue names) and candidate repressors (x-axis, red names) were selected and one (light grey), two (grey), or five (dark grey) motifs were inserted. Control ratio was based on the sequence without any motif insertions. Boxes represent 25-75% quartiles with line indicating median, whiskers extend to +/-1.5*IQR (inter-quartile range) past the boxes, and points are observations falling outside of this range. Asterisks denote p-values comparing the groups which bars connect.

*** p<=2.2E-16

** p<=1E-8

* p<=0.05

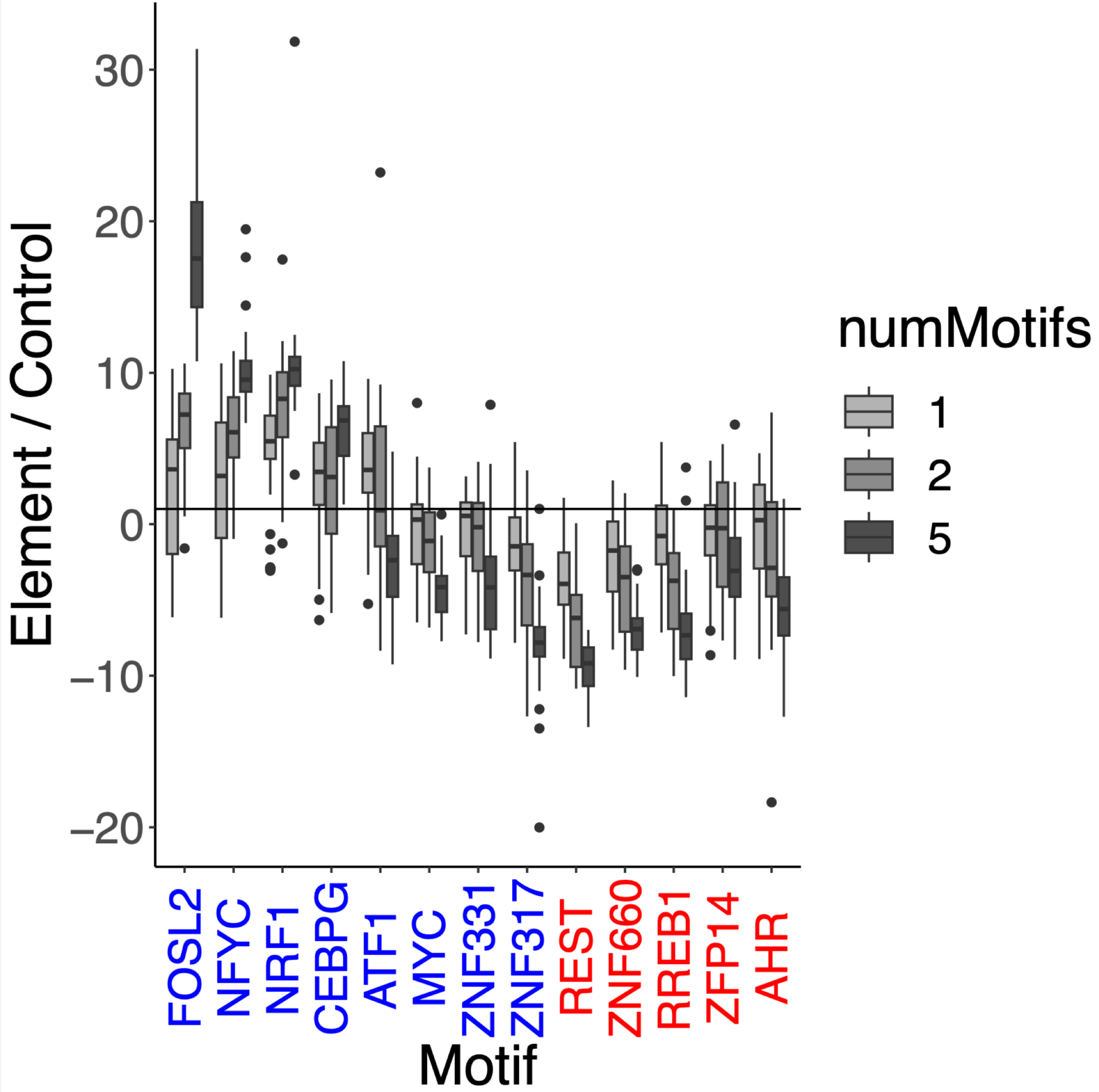

Supplemental Figure 23. Signal in lentiMPRA (natural log of normalized RNA reads over normalized DNA reads) ratio over ENH_HMM_B_1 control element signal distribution (y-axis) for motifs inserted into enhancer sequences at various intervals (x-axis). A group of candidate activators (x-axis, blue names) and candidate repressors (x-axis, red names) were selected and one (light grey), two (grey), or five (dark grey) motifs were inserted. Control ratio was based on the sequence without any motif insertions. Boxes represent 25-75% quartiles with line indicating median, whiskers extend to +/-1.5*IQR (inter-quartile range) past the boxes, and points are observations falling outside of this range. Asterisks denote p-values comparing the groups which bars connect.

*** p<=2.2E-16

** p<=1E-8

* p<=0.05

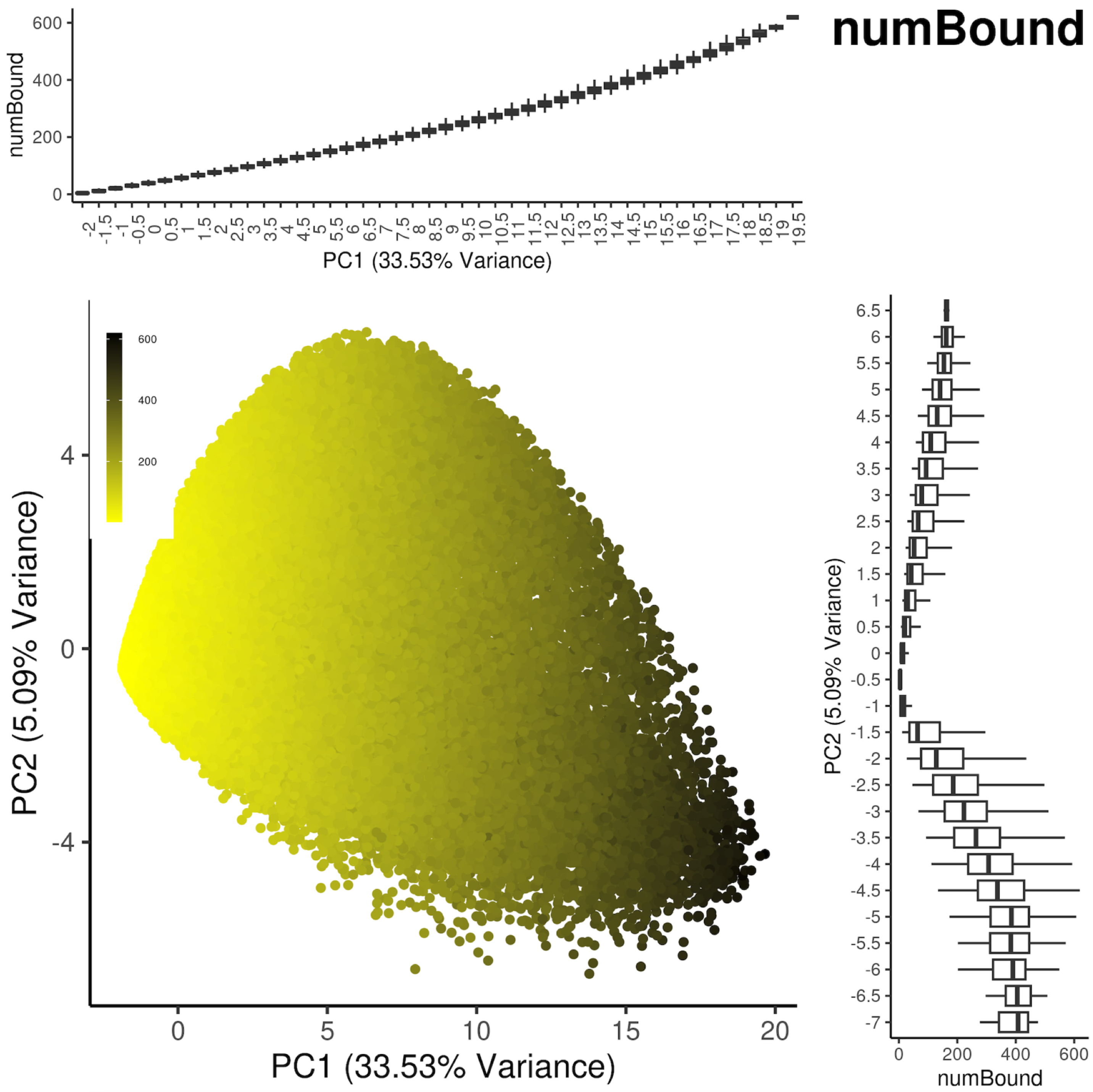

Supplemental Figure 24. Number of DAPs bound is related to the first 2 principal components. We constructed a matrix of DAPs bound across 2kb genomic bins, restricted to bins with at least 3 factors bound, and performed principal components analysis on the resulting matrix. We then plotted PC1 versus PC2, coloring points by the number of DAPs bound. **Main**: Scatterplot of PC1 and PC2 of a genome-wide DAP binding matrix, with points colored by the number of DAPs bound to a region (darker = higher number of factors bound). **Inset, top**: Boxplot of number of DAPs bound (y-axis) as a function of binned PC1 (x-axis). **Inset, right**: Boxplot of number of DAPs bound (x-axis) as a function of binned PC2 (y-axis). Boxes represent 25-75% quartiles with line indicating median, whiskers extend to +/-1.5*IQR (inter-quartile range) past the boxes.

Promoter

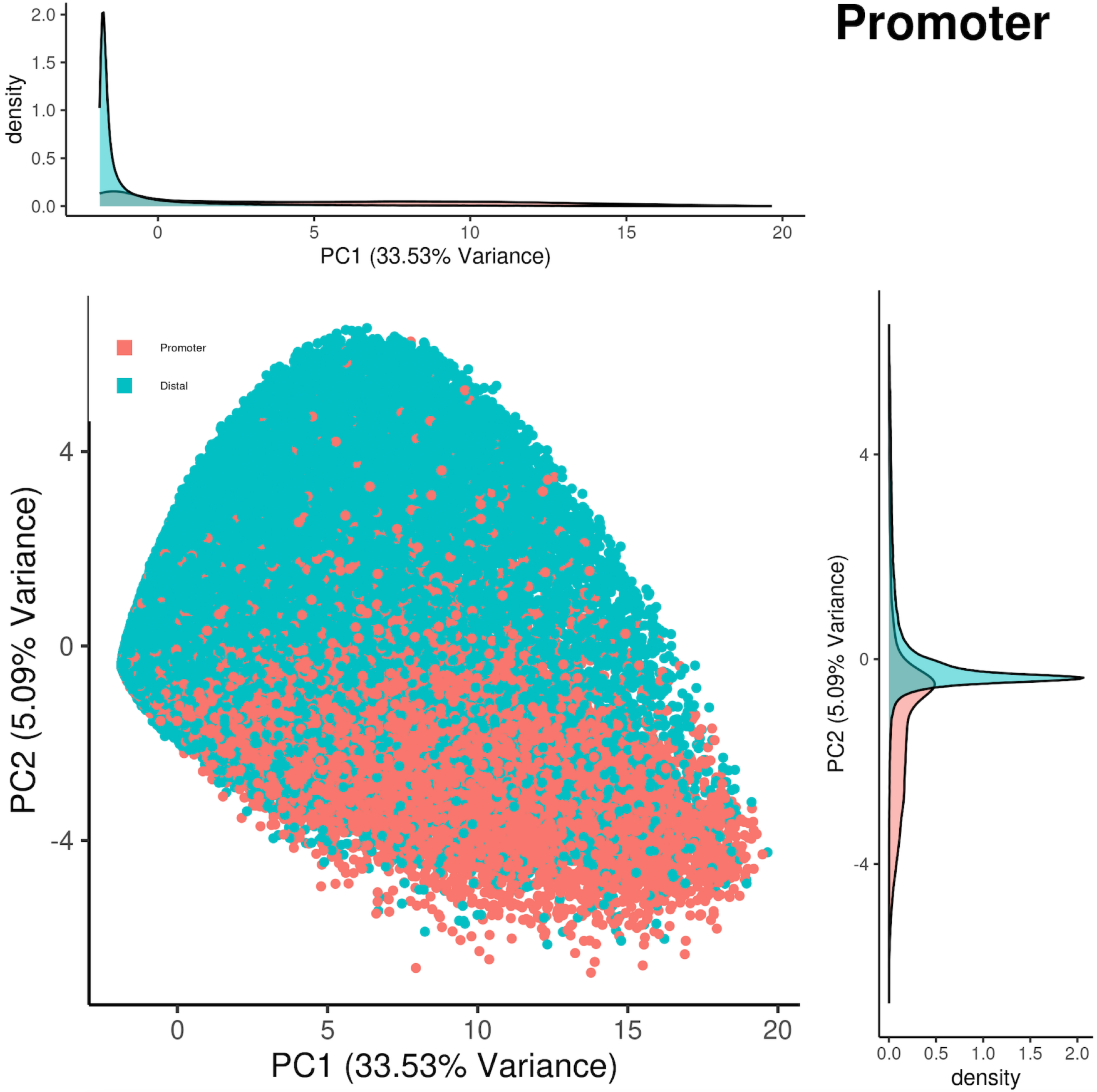

Supplemental Figure 25. Proximal versus distal identity of a region is related to the first 2 principal components. We constructed a matrix of DAPs bound across 2kb genomic bins, restricted to bins with at least 3 factors bound, and performed principal components analysis on the resulting matrix. We then plotted PC1 versus PC2, coloring points by whether a region overlapped with an annotated Promoter region. **Main**: Scatterplot of PC1 and PC2 of a genome-wide DAP binding matrix, with points colored by Promoter-proximal (+/- 1000 bp of a TSS, red) or distal (all other regions, blue). **Inset, top**: Density plot (y-axis) of promoter (red) and distal (blue) regions as a function of PC1 (x-axis). **Inset, right**: Density plot (x-axis) of promoter (red) and distal (blue) regions as a function of PC2 (y-axis).

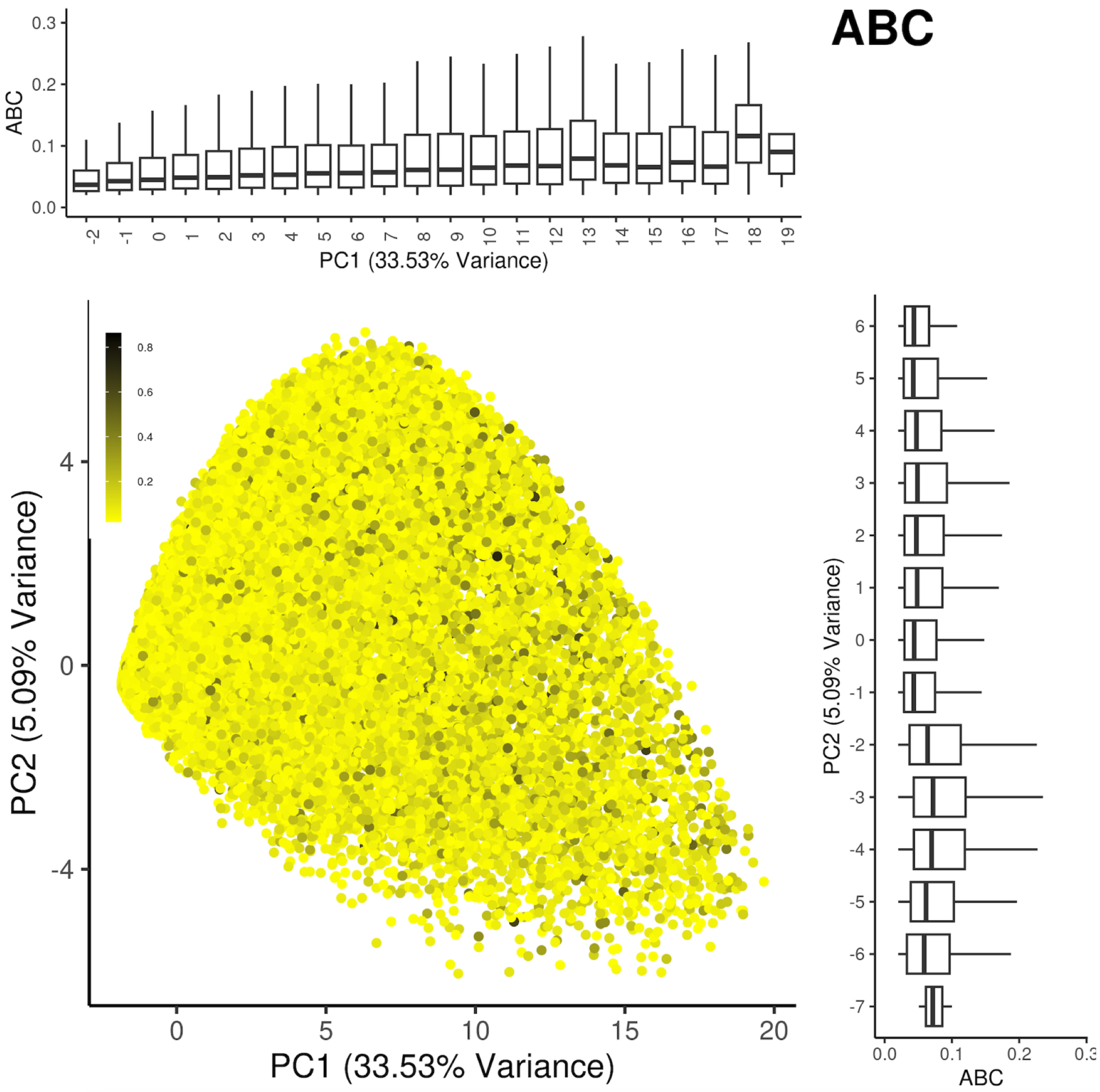

Supplemental Figure 26. Activity-by-contact intensity follows principal components. We constructed a matrix of DAPs bound across 2kb genomic bins, restricted to bins with at least 3 factors bound, and performed principal components analysis on the resulting matrix. We then plotted PC1 versus PC2, coloring points by the highest ABC score for which there was ABC support in each region. Regions without any ABC overlap are not shown. **Main**: Scatterplot of PC1 and PC2 of a genome-wide DAP binding matrix, with points colored by ABC score intensity (darker = stronger ABC). ABC score increases with PC1 (Spearman's Rho = 0.26, p <= 2.2E-16), which is highly correlated with DAPs bound, and decreases with PC2 (Spearman's Rho = -0.07, p <= 2.2E-16), which separates promoter and distal regions. **Inset, top**: Boxplot of ABC score distribution (y-axis) as a function of binned PC1 (x-axis). **Inset, right**: Boxplot of ABC score distribution (x-axis) as a function of binned PC2 (y-axis). Boxes represent 25-75% quartiles with line indicating median, whiskers extend to +/-1.5*IQR (inter-quartile range) past the boxes.

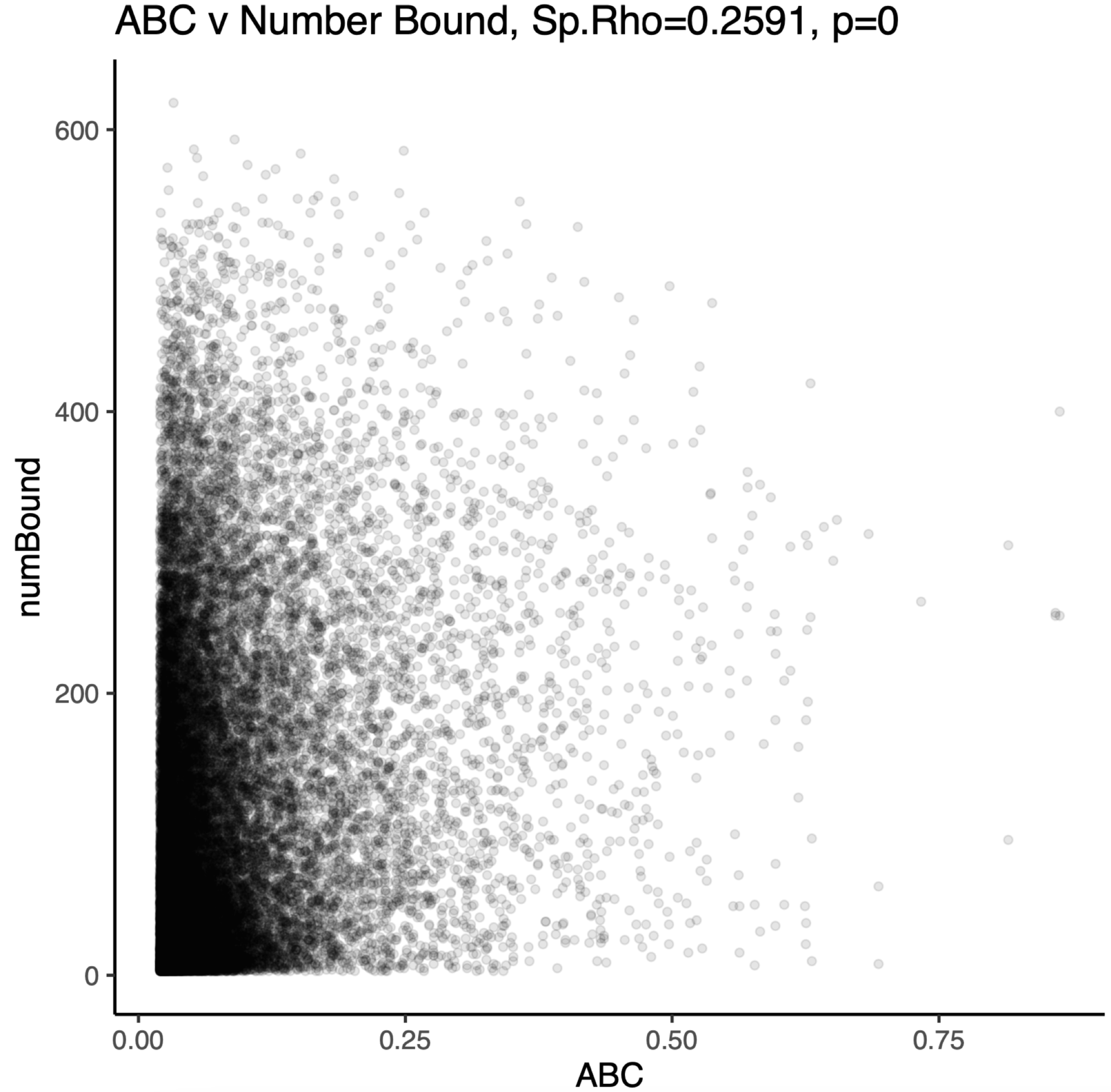

Supplemental Figure 27. Scatterplot of a region's ABC score (x-axis) versus the number of DAPs bound to the region (y-axis) for regions that have an ABC score. Spearman's Rho = 0.2591, p <= 2.2E-16.

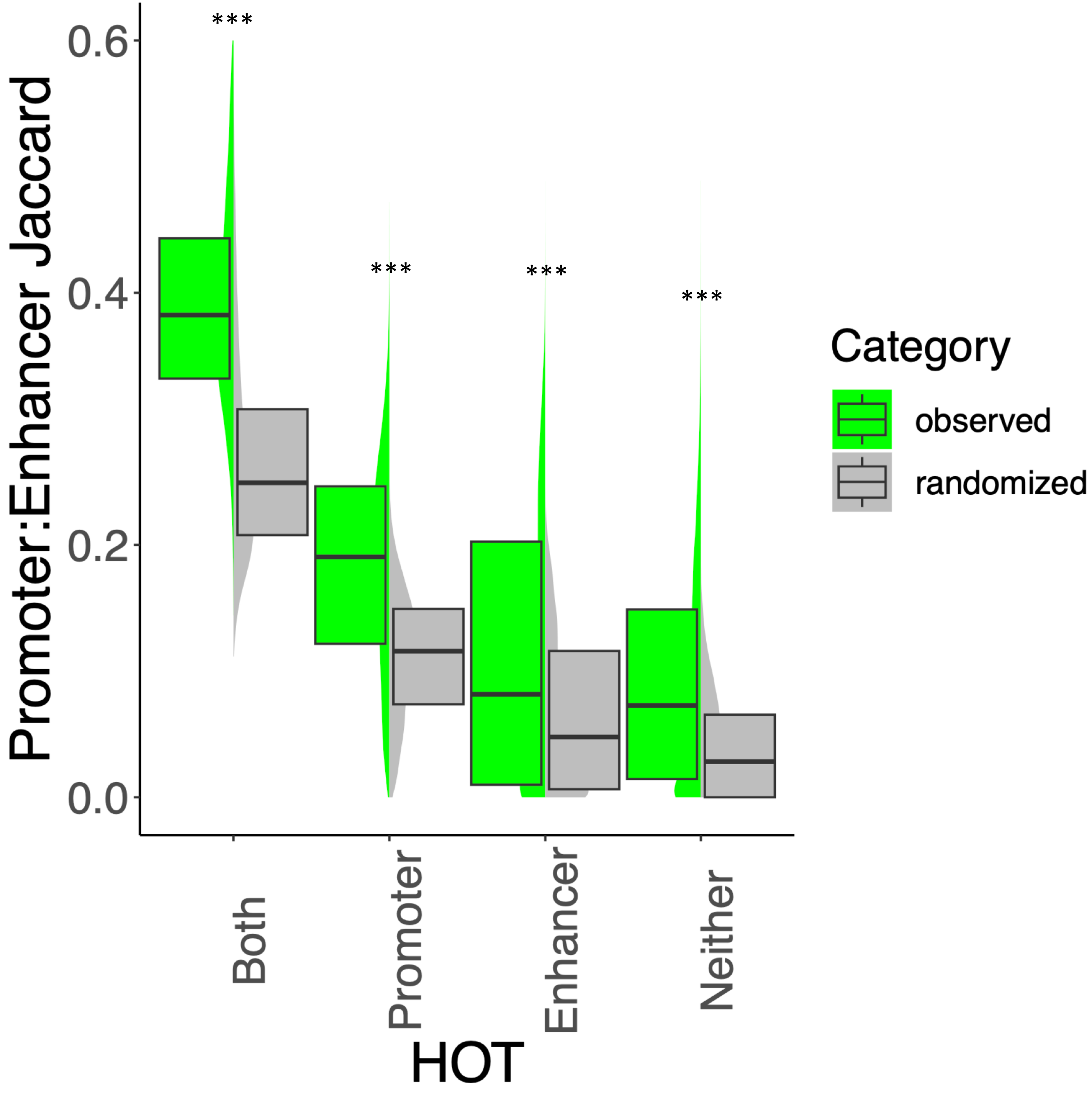

Supplemental Figure 28. Boxplot showing the Jaccard index (y-axis) of DAP identity between a promoter and distal region involved in an ABC loop when the promoter, putative enhancer (labeled “Enhancer” for plot simplicity), both, or neither are in a HOT site (x-axis). Green boxes indicate observed Jaccard index distribution, while grey boxes show Jaccard index when DAP identity is randomized. Boxes represent 25-75% quartiles with line indicating median. Density is plotted behind boxplots to show density distribution past the 25-75% IQR.

*** $p <= 2.2E-16$

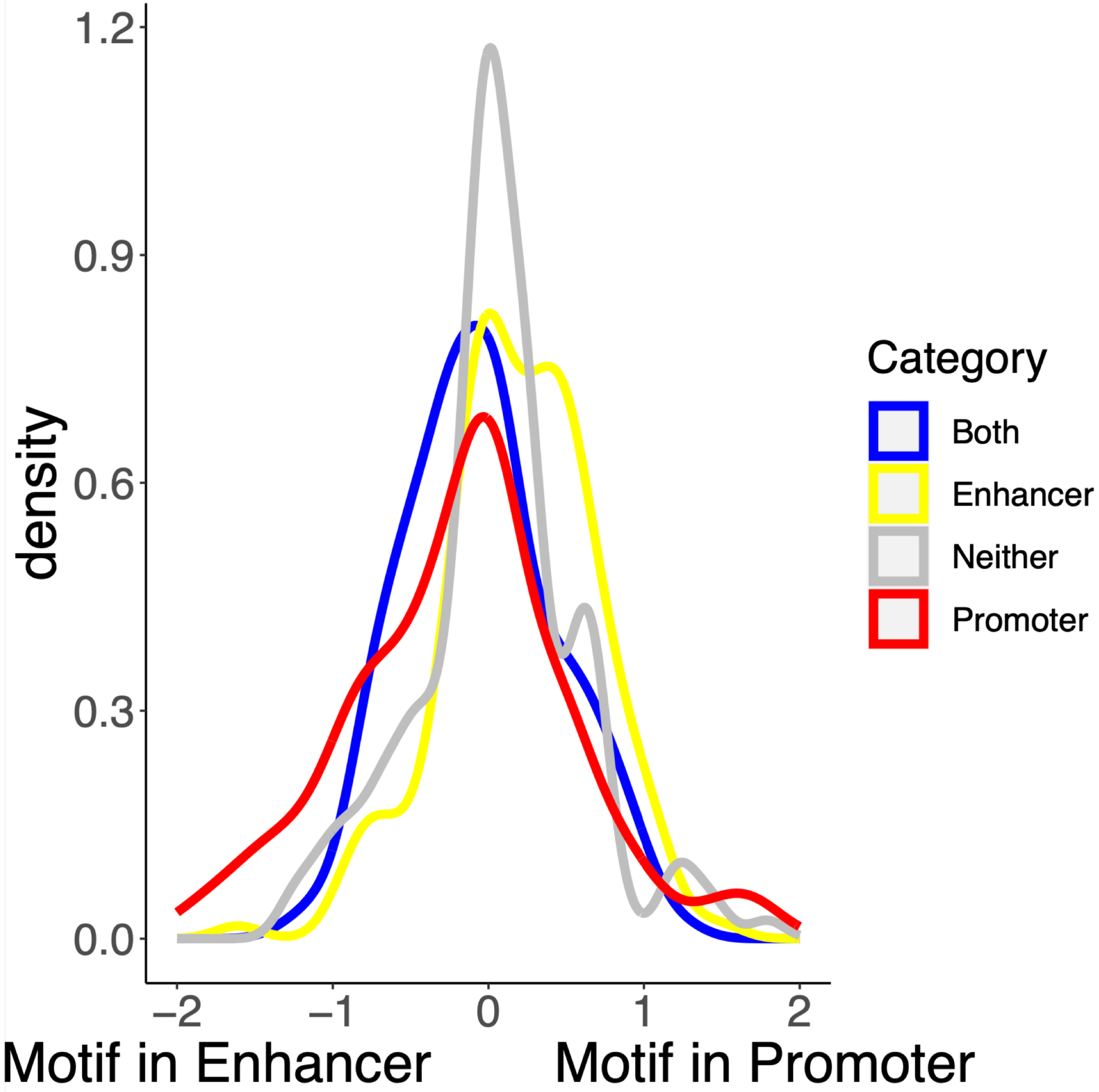

Supplemental Figure 29. Density plot of the natural log of fraction promoters with the relevant motif over fraction of putative enhancers (labeled "Enhancer" for plot simplicity) with the relevant motif for loops in which a DAP's peak is found in both the putative enhancer and the promoter, restricted to cases of promoters lacking a CpG Island. Blue indicates that both putative enhancer and promoter are HOT. Red indicates that only the promoter is HOT. Yellow indicates that only the putative enhancer is HOT. Grey denotes that neither are HOT. K-S test of Enhancer versus Promoter distribution p-value 1.44E-7.

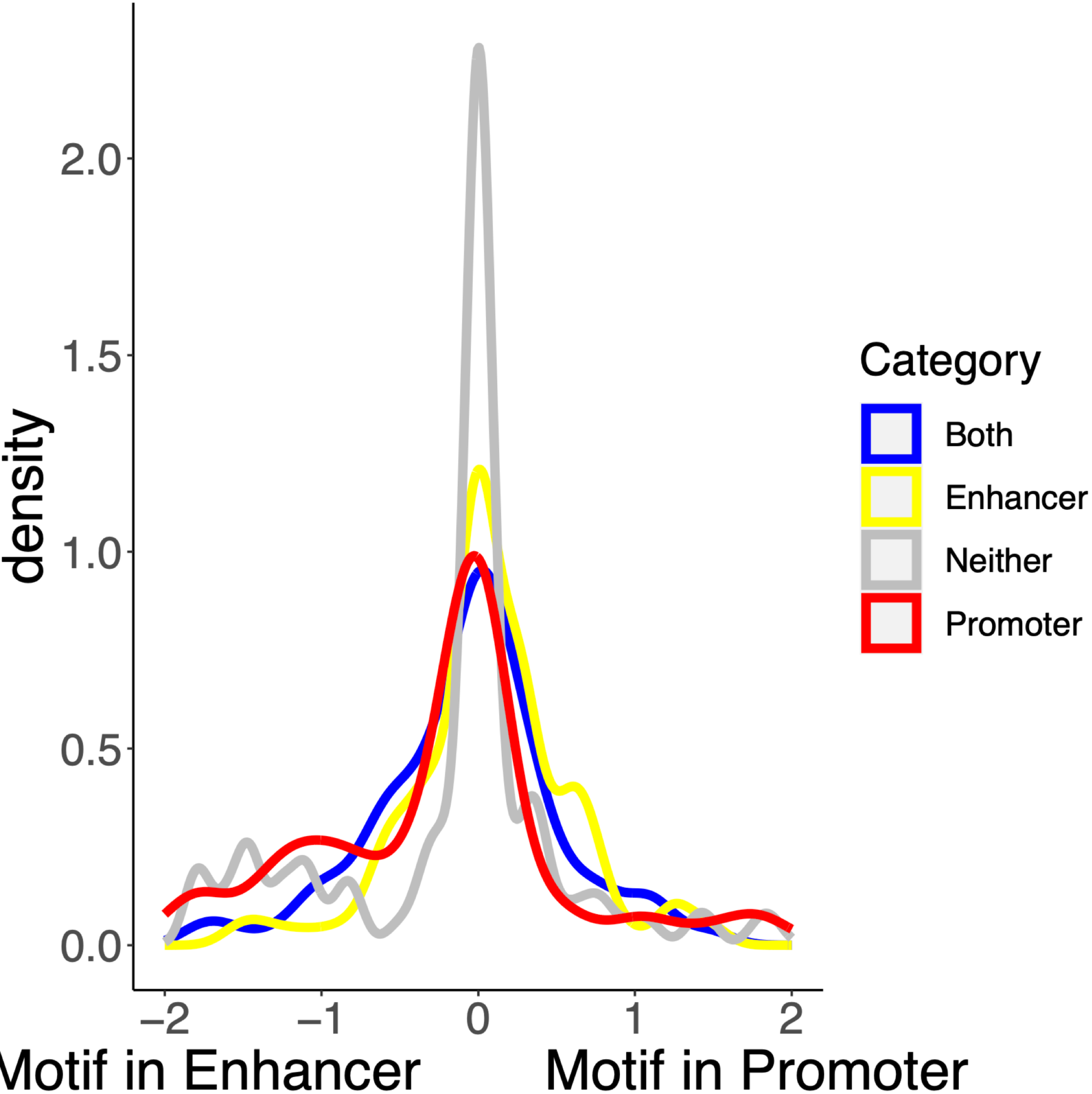

Supplemental Figure 30. Density plot of the natural log of fraction promoters with the relevant motif over fraction of putative enhancers (labeled "Enhancer" for plot simplicity) with the relevant motif for loops in which a DAP's peak is found in both the enhancer and the promoter, restricted to cases of promoters containing a constitutive CpG Island. Blue indicates that both putative enhancer and promoter are HOT. Red indicates that only the promoter is HOT. Yellow indicates that only the putative enhancer is HOT. Grey denotes that neither are HOT. K-S test of Enhancer versus Promoter distribution p-value 1.06E-4.

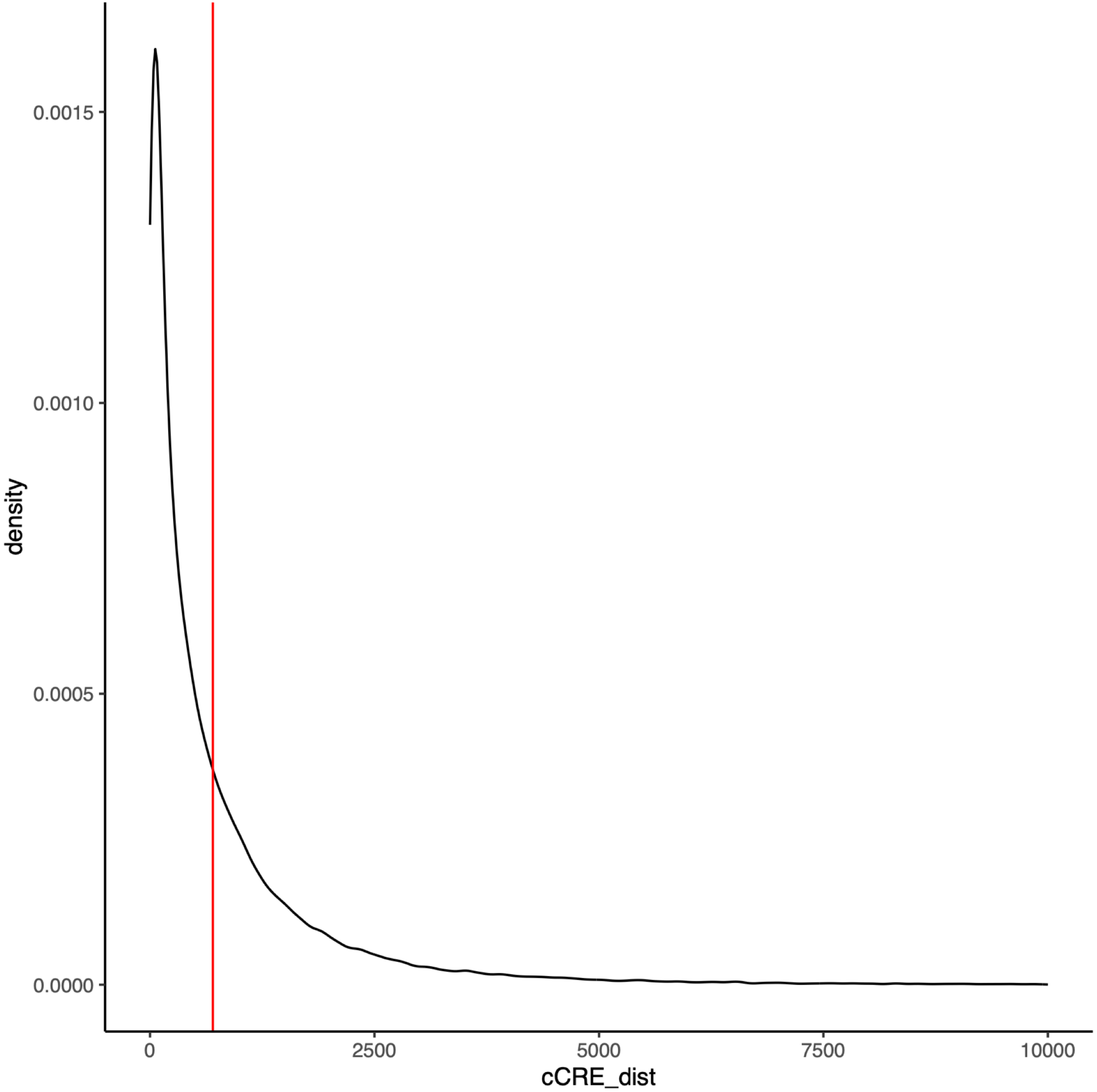

Supplemental Figure 31. Density plot of non-cCRE bound regions (y-axis) as a function of distance to nearest open-chromatin cCRE (x-axis). Red line denotes 700bp from nearest cCRE.

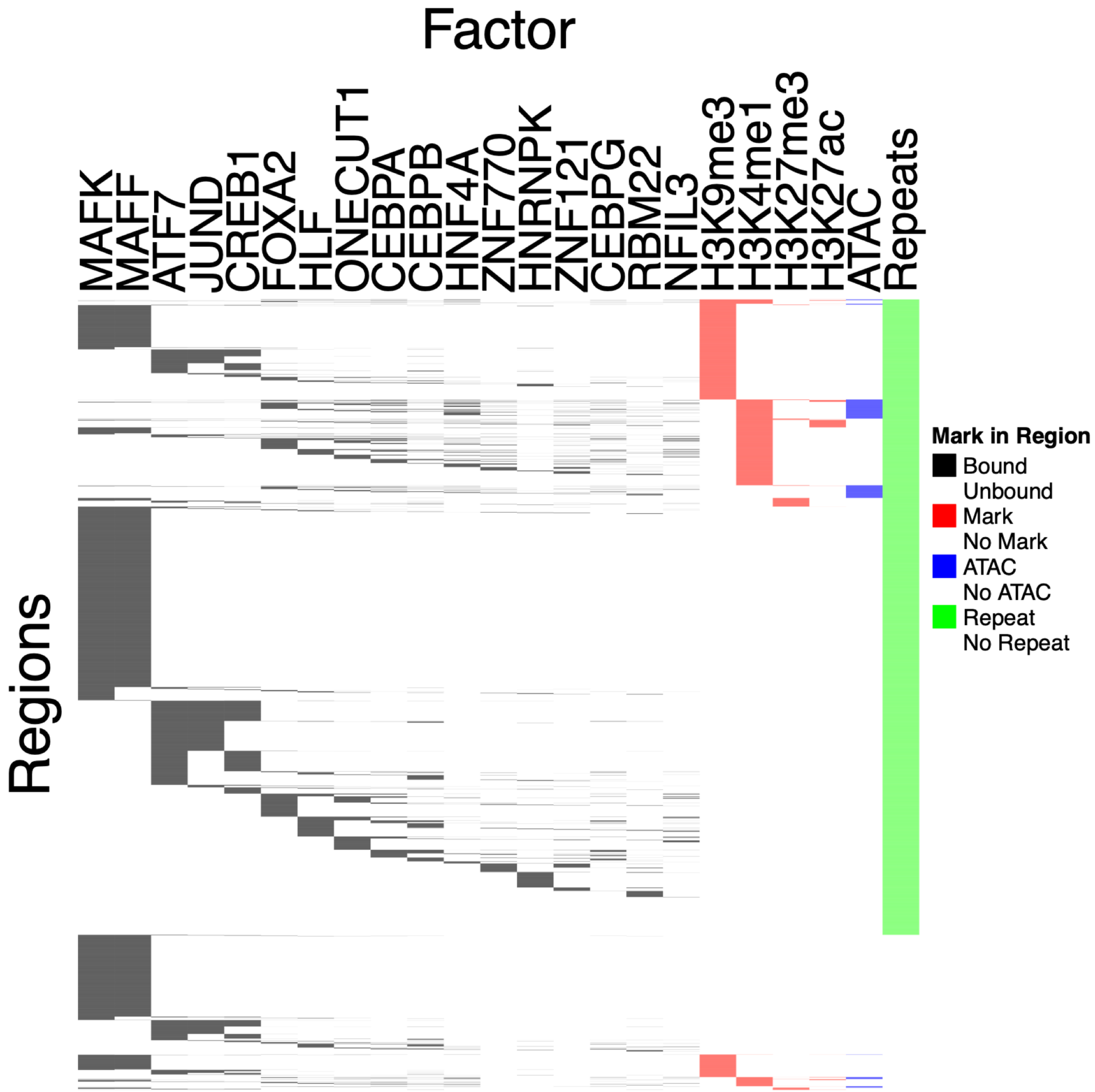

Supplemental Figure 32. Heatmap of non-cCRE regions with binding of various transcription factor and histone marks, with repetitive sequence shown in green. A majority (80%) of non-cCRE bound regions fall in repetitive sequence.

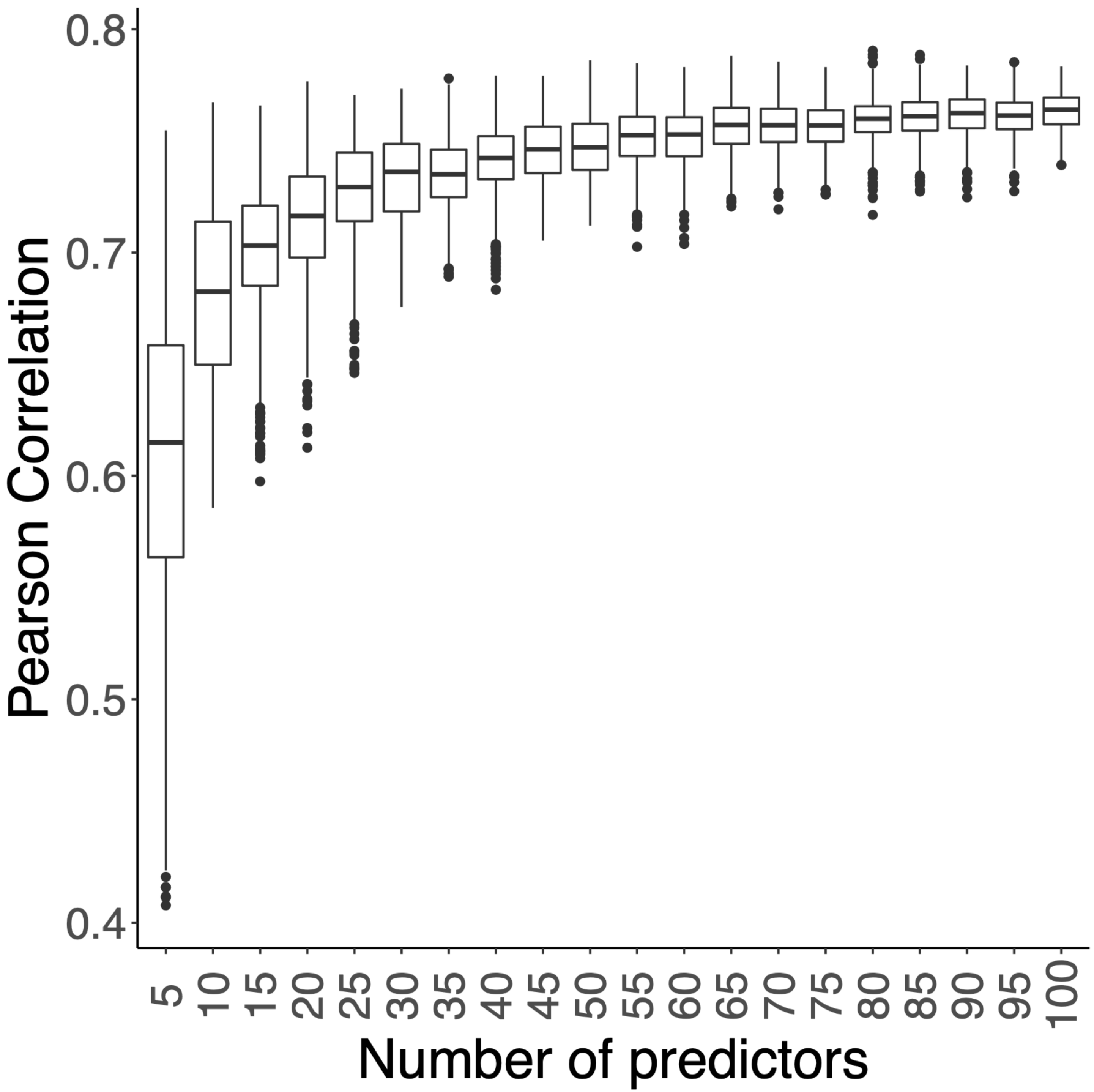

Supplementary Figure 33. Boxplot shows model prediction accuracy distributions (y-axis) as measured by the Pearson's correlation coefficient for models of TF binding on gene expression, binned by the number of TFs used to build the model (x-axis). Boxes represent 25-75% quartiles with line indicating median, whiskers extend to +/-1.5*IQR (inter-quartile range) past the boxes, and points are observations falling outside of this range.

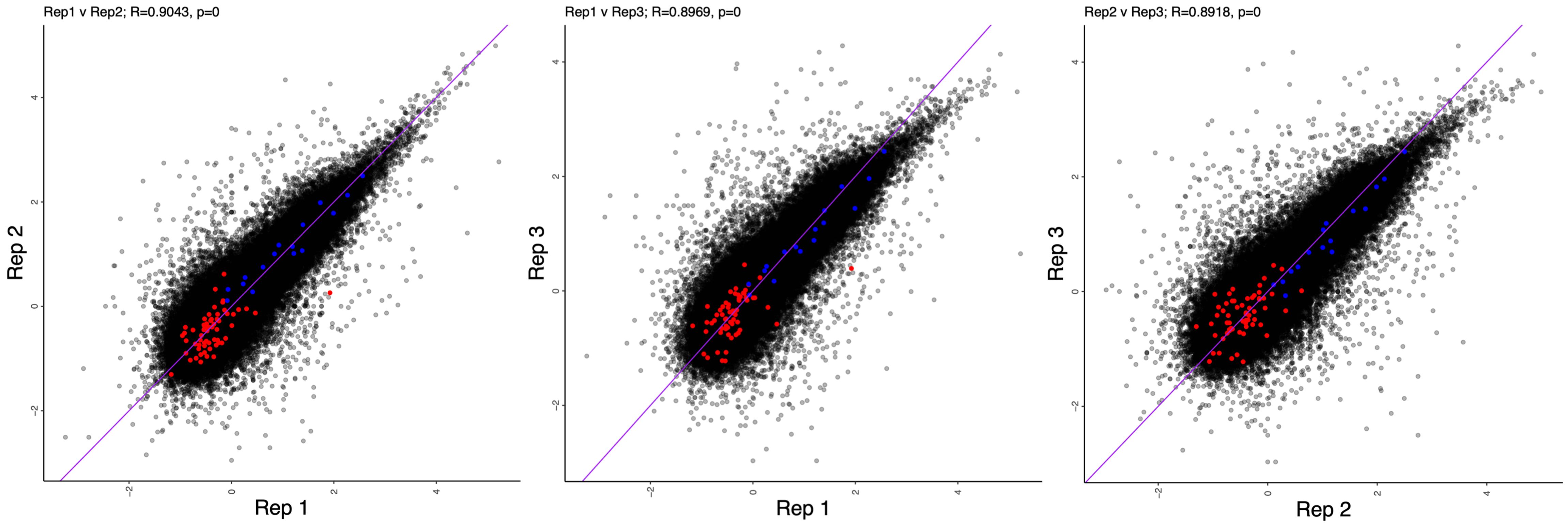

Supplemental Figure 34. Scatterplots showing signal in lentiMPRA (natural log of normalized RNA reads over normalized DNA reads) compared across replicates for locally-performed lentiMPRA. Red points denote negative controls, blue points denote positive controls. **Left**: Rep1 versus Rep2. **Middle**: Rep1 versus Rep3. **Right**: Rep2 versus Rep3.

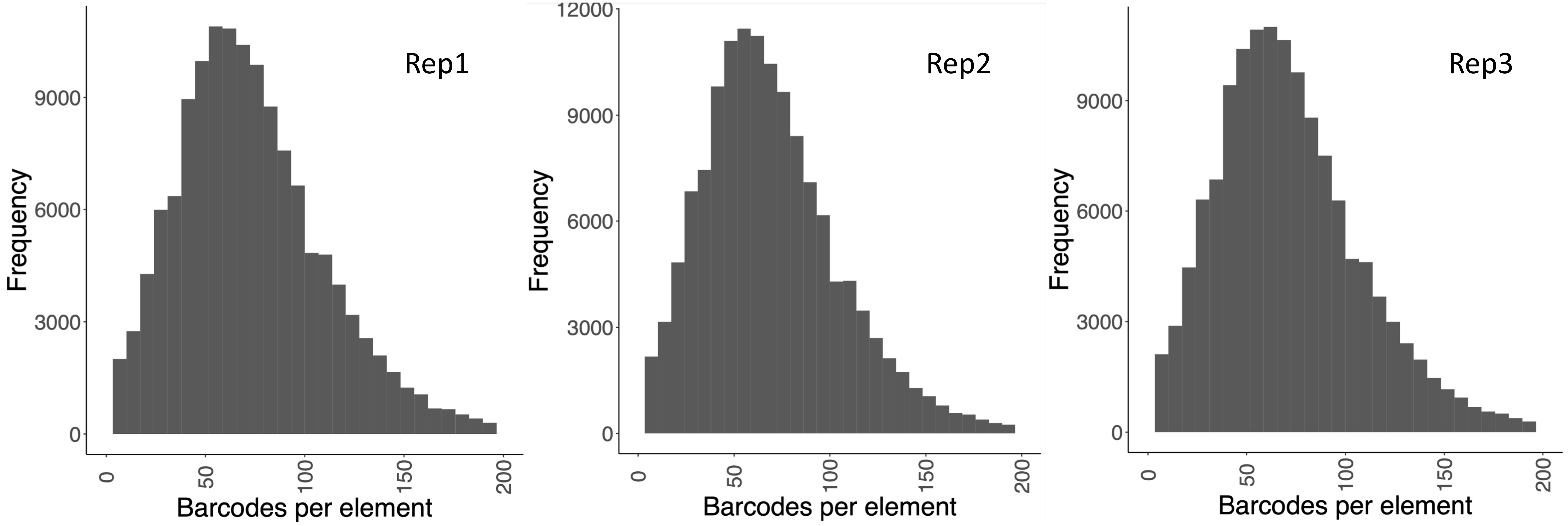

Supplemental Figure 35. Barplot showing the number of elements (y-axis) as a function of the observed number of binned barcodes per element (x-axis). **Left:** Replicate 1. **Middle:** Replicate 2. **Right:** Replicate 3.
